# Global m6A RNA and whole 5mC DNA methylation specifically contribute to cell replicative and premature senescence induced by extrinsic oxidative stress

**DOI:** 10.1128/msystems.01647-24

**Published:** 2025-06-16

**Authors:** Chenyu Zhu, Tingting Huang, Jiaqi Fu, Min Li, Luyi Tan, Xinyu Zhang, Wenli Cheng, Caiyun Lai, Zhangying Wang, Wenji Zhang, Wenjuan Zhang

**Affiliations:** 1 Department of Public Health and Preventive Medicine, School of Medicine, Jinan University 727092 , Guangzhou, Guangdong, China; 2 Key Laboratory of Crop Genetic Improvement of Guangdong Province, Crops Research Institute, Guangdong Academy of Agricultural Sciences 714036, Guangzhou, Guangdong, China; University of California San Diego, La Jolla, California, USA

**Keywords:** H_2_O_2_, N6-methyladenosine, 5-methylcytosine, premature senescence, epigenetics

## Abstract

Hydrogen peroxide (H_2_O_2_) is a typical representative substance of environmental oxidative stress. Exogenous substances can alter epigenetic modifications from the DNA to the RNA level through oxidative stress. We investigated methylation profiles of whole RNA m6A and DNA 5mC in the epitranscriptome and epigenome of human lung embryonic fibroblasts in replicative senescence and H_2_O_2_-induced premature senescence. By RNA-seq, the expression of most RNA m6A and DNA 5mC regulators was reduced in both replicative and premature senescence respectively, whereas the expression of most senescence-associated secretory phenotypes, such as SASP, was increased in premature senescence. MeRIP-seq revealed that RNA m6A methylation sites were relatively conserved in replicative and premature senescence, but that premature senescence had higher levels of m6A methylation than replicative senescence. MeDIP-seq results showed that 5mC methylation was higher in replicative senescence than in premature senescence, and the methylation peak with the largest difference appeared on chromosome 19. DO enrichment analysis indicated that RNA m6A methylation played a key role in malignant tumor regulation in replicative senescence, whereas DNA 5mC promoted malignant tumor in premature senescence. Next, to explore the interaction of RNA m6A and DNA 5mC in the senescent state, we screened common hub genes for replicative and premature senescence. Four m6A-modified target genes, namely, *ASPM*, *CENPF*, *MKI67*, and *BLM*, were all closely associated with mitosis and cell cycle regulation. In addition, we also screened 5mC target genes including *CDC45*, *TPX2,* and *UBE2T*.

**IMPORTANCE:**

RNA-seq showed that most of the m6A and 5mC regulators and the majority of SASP expression were downregulated. The m6A motif was conserved in replicative and premature senescence, and its methylation was higher in replicative senescence. The most differentially 5mC methylation peak was located on chromosome 19, and its methylation was higher in premature senescence. Gene regulation by m6A in replicative senescence and 5mC in premature senescence was enriched in malignant tumors.

## INTRODUCTION

Cell senescence is a primary risk factor for age-related diseases, exerting profound physiological and pathological effects on human tissues and organs. Various stressors, environmental factors, and lifestyle choices can induce irreparable cellular damage, extracellular matrix dysfunction, and disruptions in intercellular communication and signaling. These alterations ultimately contribute to the development of age-related diseases and mortality (1, 2). Cell senescence can arise from chronic or acute injury, leading to replicative senescence and stress-induced premature senescence (3). Both types are hallmarks of organismal aging, characterized by irreversible cell cycle arrest and distinct phenotypic changes (4). Replicative senescence is primarily driven by telomere shortening and activation of the DNA damage response pathway due to repeated cell division, culminating in p53 activation in the absence of external stimuli. In contrast, premature senescence is typically triggered by oxidative stress, ionizing radiation, or cytotoxic therapies, leading to cell cycle dysregulation, including activation of the cyclin-dependent kinase (CDK) inhibitor 2A pathway. Despite their distinct triggers, both types of senescence activate the cyclin-dependent kinase inhibitor p21^CIP1^, inducing permanent cell cycle arrest (5). The divergent regulatory mechanisms underlying replicative and premature senescence present unique opportunities for targeted interventions.

Epigenetic alterations play a pivotal role in driving cellular senescence, influencing health span and disease susceptibility (6, 7). Both intrinsic and external factors modulate the expression of key epigenetic marks, including DNA 5-methylcytosine m(5mC) methylation , RNA N6-methyladenosine (m6A) methylation, A-to-I RNA editing, and noncoding RNAs, thereby regulating senescence progression (8). DNA methylation, one of the most common epigenetic modifications, serves as a critical link between environmental changes and cell responses, with 5mC being ubiquitous in the human genomes (9). Modifications in DNA methylation patterns are strongly associated with age-related diseases, including ulcerative colitis, atherosclerosis, type II diabetes, and Alzheimer’s disease (10–13). Suppression of the 5mC methyltransferase DNA methyltransferase 2 (DNMT2) induces oxidative stress, genomic instability, and altered expression of proliferation-associated miRNAs, impairing cell growth and accelerating senescence (14). Moreover, distinct DNA methylation patterns at specific loci and CpG sites have been observed in senescent human fetal lung fibroblasts and foreskin fibroblasts, suggesting cell-type-specific epigenetic regulation during aging (15). The m6A modification, the most abundant internal mRNA modification in eukaryotes, plays a crucial role in cellular senescence via reactive oxygen species (ROS)-mediated pathways. Dysregulation of m6A-related enzymes, binding proteins, and targeted genes have been linked to age-related neurodegenerative diseases (16). Abnormal m6A methylation is associated with age-related neurodegenerative diseases, including Parkinson’s and Alzheimer’s disease, as well as age-related infertility and heart failure in murine models (17, 18). Methyltransferase-like protein 3 (METTL3), a key m6A methyltransferase, is closely associated with senescence (19). Its knockdown accelerates senescence in human mesenchymal stem cells (20). Reduced METTL3 expression has been observed in senescent human diploid fibroblasts and human renal tubular epithelial cells, and overexpression of the methyltransferase-like protein 14 (METTL14) attenuated senescence in normal and Hutchinson-Gilford progeria syndrome cells, and alleviated endothelial cell senescence in a YTHDF2-dependent manner (21, 22).

Although 5mC and m6A are distinct epigenetic modifications targeting DNA and RNA, respectively, they interact through complex cross-regulatory networks to collectively modulate the aging processes. Functionally, these modifications cooperatively regulate critical pathways in cellular senescence. For instance, combined inhibition of DNMT1 and METTL3 not only synergistically reduces cell cycle arrest in hepatocellular carcinoma cells but also suppresses tumor immune evasion via downregulation of inflammatory signaling pathways (23). These findings reveal a functional coordination between the two modification systems in governing cell fate decisions. At the mechanistic level, they establish an interdependent regulatory circuit. Specifically, METTL3-mediated m6A methylation facilitates the TET1 mRNA stability and translation, thereby augmenting 5mC demethylation activity. Conversely, TET1-mediated DNA demethylation reciprocally regulates METTL3 transcription, thereby establishing a feedback loop that orchestrates global m6A methylation dynamics (24). In terms of target gene regulation, DNA methylation directly modulates the expression of the critical senescence gene p16INK4a through DNMT-mediated promoter methylation (25). Meanwhile, m6A modification indirectly suppresses the expression of cell cycle inhibitors, such as p16INK4a and p21CIP1, by regulating the stability of PEG10 mRNA, thereby establishing a dual regulatory mechanism that collectively promotes cell cycle progression (26). In relation to the oxidative stress response, TET1-mediated regulation of the 5mC methylation state of the GSTP1 promoter exerts protection against arsenic-induced oxidative stress in human bronchial epithelial cells (27). On the other hand, METTL3-mediated m6A modification regulates the mRNA stability of glutathione peroxidase 4, thereby inhibiting ferroptosis and enhancing epithelial cell viability. This leads to a reduction in airway inflammation in a mouse asthma model (28).

Epigenetic mechanisms provide critical insights into the initiation and progression of aging and related diseases. Environmental pollutants can trigger the production of ROS, and an imbalance between ROS generation and detoxification leads to oxidative damage, resulting in the accumulation of macromolecule damage. Hydrogen peroxide (H_2_O_2_), a potent oxidant, induces premature senescence by generating excessive ROS (29). Similar to replicative senescence, H_2_O_2_-induced premature senescence exhibits hallmark features, including increased senescence-associated β⁃galactosidase (SA-β⁃gal) activity, decreased proliferative capacity, cell cycle arrest, and increased expression of senescence-associated proteins, with distinct molecular mechanisms (30). Our previous studies using an H_2_O_2_-induced premature senescence model demonstrated that m6A methylation regulates cell senescence via key targets SIRT3, IRS2, and E2F3 (16). Yet, comprehensive 5mC and m6A methylome profiles remain uncharacterized, and it is unclear whether their methylation patterns and genomic distributions differ between senescence types.

In this study, we employed methylated DNA immunoprecipitation sequencing (MeDIP-seq), methylated RNA immunoprecipitation sequencing (MeRIP-seq), and high-throughput RNA sequencing (RNA-seq) to delineate the genome-wide 5mC and m6A methylation landscapes in both replicative and H_2_O_2_-induced premature senescence. Our findings reveal distinct regulatory mechanisms of 5mC and m6A in cell senescence and identify dual-regulated target genes, offering novel insights for clinical intervention and early prevention of aging-related diseases.

## MATERIALS AND METHODS

### Cell culture and treatment

Human embryonic fibroblast(HEFs) were obtained from the Cell Resource Center, Institute of Basic Medical Sciences, Chinese Academy of Medical Sciences, and cultured aseptically in low-sugar Dulbecco’s modified Eagle medium (L-DMEM, Gibco, Grand Island, NY, USA) with 10% fetal bovine serum, 100 U/mL penicillin, and 0.1 mg/mL streptomycin (Gibco, Grand Island, NY, USA) in sterile culture. Constant temperature incubator (Thermo Fisher, Waltham, MA, USA) conditions were strictly controlled at 37°C, 95% relative humidity, and 5% CO_2_. HEFs were continuously passaged and stopped proliferating at around 52 population doubling levels (PDL) and showed senescent expression. Based on the age definition of the cell culture, we classified them into young cells (22PDL) and replicative senescent cells (49PDL). HEFs around 22PDL were exposed to 400 H_2_O_2_ µmol/L (BDH Chemicals Ltd., Poole, UK) for 2 h per day for 4 days, and then given fresh L-DMEM with fetal bovine serum and cultured for another 7 days, defined as the premature senescence persistence group (PSp), as previously described (31).

### RNA-seq and data analysis

Total RNA was extracted using Trizol reagent (Invitrogen, Grand Island, NY, USA) and quantified by NanoDrop ND-1000 spectrophotometer (Thermo Fisher Scientific, Waltham, MA, USA). Total RNA (1 µg) was used for removing the rRNAs using Ribo-Zero rRNA Removal Kits (Illumina, San Diego, CA, USA). The rRNA was removed using Ribo-Zero rRNA Removal Kits (Illumina), and libraries were prepared with the TruSeq Stranded Total RNA Library Prep Kit (Illumina). Sequencing was performed on an Illumina HiSeq Sequencer (150 cycles). After quality control, reads were aligned to the reference genome (UCSC MM10) using Hisat2. Differentially expressed mRNAs (*P* < 0.05, fold change ≥ 2) were identified using Cuffdiff. Gene Ontology (GO) and Kyoto Encyclopedia of Genes and Genomes (KEGG) analyses were performed for functional annotation.

### MeRIP-seq and data analysis

Total RNA was prepared as in RNA-seq. MeRIP-Seq was performed by Cloudseq Biotech Inc. (Shanghai, China) according to the procedure in the reagent kit instructions. Briefly, m6A RNA immunoprecipitation was performed using the GenSeqTM m6A RNA IP Kit, and libraries were generated with the NEBNext Ultra II Directional RNA Library Prep Kit. Sequencing was performed on an Illumina HiSeq instrument (150 bp paired-end). Data were aligned (Hisat2, UCSC MM10), and methylated sites were identified using MACS. Differentially methylated sites (*P* ≤ 0.0001, Fold change ≥2, diffReps) were analyzed with GO and KEGG (*P* < 0.05). IGV visualized the alignment of differentially methylated sites (DMS).

### MeDIP-seq and data analysis

DNA samples were fragmented (200–800 bp) with a Diagenode Bioruptor and sequenced on an Illumina HiSeq 4000. The libraries were then sequenced on the Illumina HiSeq 4000 following the HiSeq 3000/4000 SBS Kit (300 cycles) protocol. After sequencing images were generated, the stages of image analysis and base calling were performed using Off-Line Basecaller software (OLB V1.8). After passing the Solexa CHASTITY quality filter, clean reads were aligned (Hisat2, UCSC hg19), and peaks were called using MACS2 (*q* < 10⁻⁵). Differentially methylated regions (DMRs) were identified (|log_2_^FC^| ≥ 1, *P* ≤ 0.0001, diffReps). GO and KEGG analyses were performed (*P* < 0.05).

### Disease ontology (DO) analysis and protein–protein interaction network construction and hub gene screening

DO analyses were performed using the “DOSE” package (version 4.4) to identify disease-gene associations. The top 20 relevant diseases with the highest significance were visualized using box plots and bubble plots, and the intersection between the diseases was shown using upset and network plots. Protein-protein interaction (PPI networks were constructed using STRING (confidence >0.4) (https://cn.string-db.org/cgi/input.pl), and hub genes were identified using the MCC method in Cytoscape (MCC ≥8).

### Correlation analysis and Western blot for protein level

Based on the sequencing results, we performed a correlation analysis of the expression levels of target genes across different groups and visualized the findings using a heatmap. After being cleaned in PBS, the cells were harvested. Using a protease inhibitor called PMSF (Beyotime, Shanghai, China), the whole protein was extracted using RIPA Lysis Buffer. Using 10% SDS-polyacrylamide gel electrophoresis (SDS-PAGE, Beyotime, Shanghai, China), proteins from entire lysates were isolated and subsequently transferred individually to polyvinylidene difluoride membranes (Millipore Corporation, Burlington, MA, USA). The primary antibodies for anti-BLM (Santa Cruz, sc-365753, 1:1,000), anti-Ki-67 (Santa Cruz, sc-23900, 1:1,000), anti-CENPF (Bioswamp, PAB47268 1:1,000), and anti-ASPM (Bioswamp, PAB46848, 1:1,000) were incubated with the bolts for 1 h after they had been incubated with 5% nonfat milk. Using chemiluminescent HRP substrate (Millipore, MA, USA) and an automated chemiluminescence imaging analysis system (Tanon-5200, Beijing, China), proteins in the membrane were identified.

### Statistical analysis

The data in this study were analyzed using an unpaired Student’s t-test for comparisons between two groups and analysis of variance (ANOVA) for comparisons among multiple groups. Each group was set up with *n* = 3 replicates in the Western blot. Statistical tools included R (v4.1.0), GraphPad Prism (v8.0), and Microsoft Excel 2016. Significance was set at *P* < 0.05.

## RESULTS

### Transcriptome profiles in premature and replicative senescence

**Fig 1:**
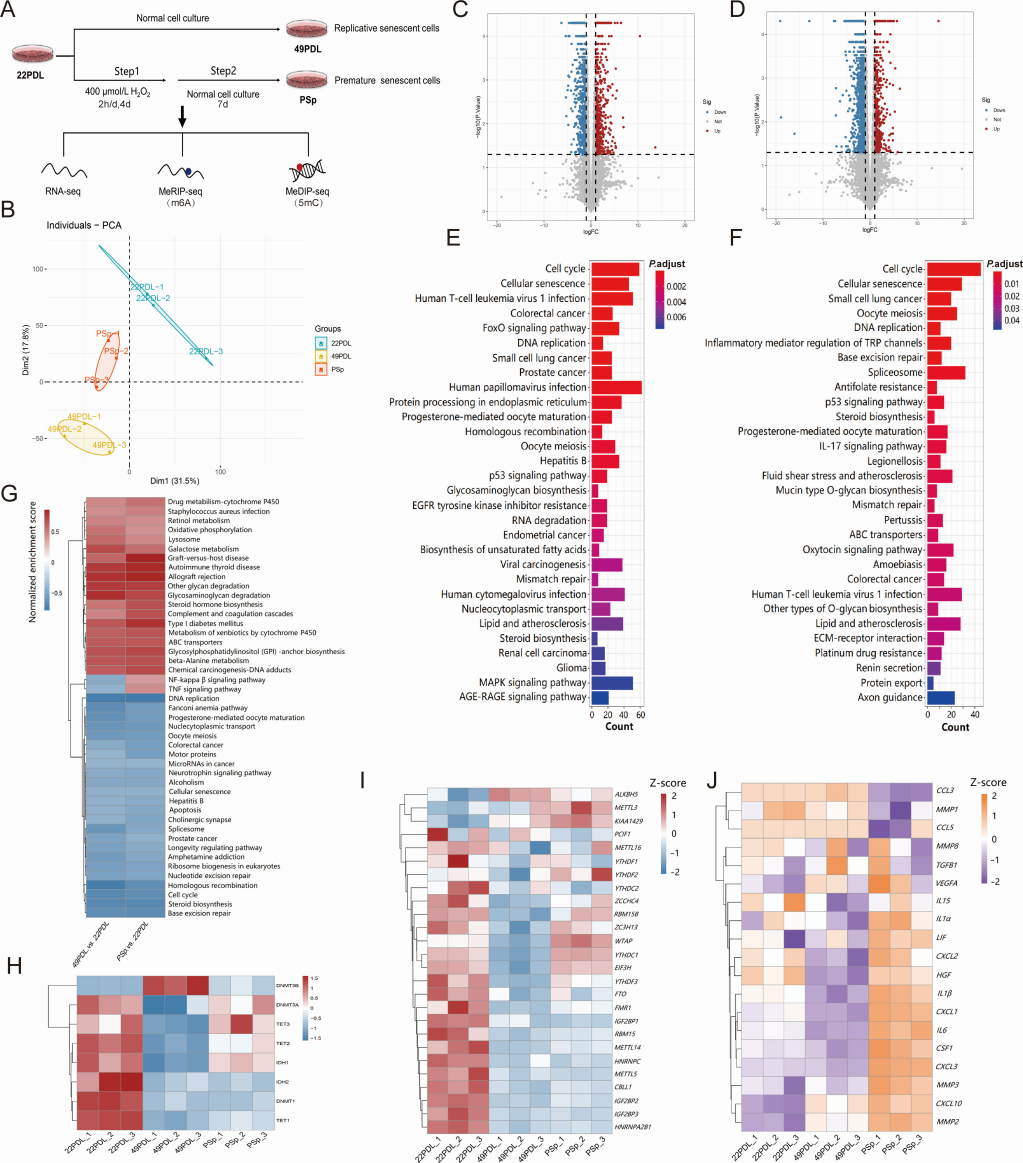
Transcriptional signatures and regulators of replicative and premature senescence. (**A**) Schematic design of the multi-omics analysis of replicative and H _2_O_2_-induced premature senescence. Human lung fibroblasts were used, and samples were collected at young (22PDL), replicative senescent (49PDL), and H_2_O_2_-induced premature senescence (PSp) for RNA-seq, MeRIP-seq (m6A), and MeDIP-seq (5mC), respectively. (**B**) PCA of the transcriptome of 22PDL, 49PDL, and PSp groups (*n* = 3). (**C, D**) Volcano plots showing DEGs in 49PDL and PSp groups. Fold change ≥ 2 and *P* < 0.05. Upregulated genes are colored in red and downregulated genes are colored in blue. (**E**) Kyoto Encyclopedia of Genes and Genomes (KEGG) pathway enrichment results for DEGs in replicative senescence. (**F**) KEGG pathway enrichment results for DEGs in H _2_O_2_-induced premature senescence. (**G**) Gene set enrichment analysis (GSEA) showing changes in common pathways of replicative and H _2_O_2_-induced premature senescence. (**H**) Heatmap showing changes in expression levels of DNA 5mC regulators (*n* = 3). (**I**) Heatmap showing changes in expression levels of RNA m6A regulators (*n* = 3). (**J**) Heatmap showing changes in expression levels of SASP-related genes (*n* = 3).

Figure 1A illustrates the experimental design of this study, including the cell groups (22PDL, 49PDL and PSp) and the corresponding key experimental procedures.Global gene expression profiles, generated through principal component analysis (PCA) of RNA-seq data, showed significant differences among 22PDL, 49PDL, and PSp groups (Fig. 1B). We determined the extent of altered mRNA expression for the 49PDL and PSp groups. Compared with the 22PDL group as a control, the differentially expressed genes (DEGs) in the 49PDL group included 574 upregulated genes and 1,872 downregulated genes (Fig. 1C). In contrast, the DEGs in the PSp group included 639 upregulated genes and 977 downregulated genes (Fig. 1D). Furthermore, GO and KEGG enrichment analyses suggested that DEGs in both 49PDL and PSp groups were enriched in several key pathways, including cell cycle, cellular senescence, DNA replication, and p53 signaling pathway (Fig. 1E and F). To confirm the transcriptome signature of replicative and H_2_O_2_-induced premature senescence, we also used gene set enrichment analysis (GSEA). Consistent with KEGG results, we found that cell cycle, DNA replication, cellular senescence, and longevity regulatory pathways were significantly downregulated in both senescence types. The FoxO pathway was significantly downregulated in replicative senescence, while the IL-17 signaling pathway, HIF-1 signaling pathway, and TGF-β signaling pathway were significantly upregulated in H_2_O_2_-induced premature senescence (Fig. S1). All these crucial pathways are closely related to cellular senescence. Notably, genes associated with replicative and H_2_O_2_-induced premature senescence were enriched in all 45 pathways. Inflammation-related pathways, such as the NF-kappa β signaling pathway and TNF signaling pathway, were downregulated in replicative senescence but upregulated in H_2_O_2_-induced premature senescence (Fig. 1G).

We performed transcriptional analysis of 25 m6A regulators and 8 5mC regulators respectively. The expression of the m6A demethylase *ALKBH5* was found to be increased in replicative and H_2_O_2_-induced premature senescence, and the expressions of some m6A methylation regulators and binding proteins were decreased in both aging processes, while *WTAP*, *YTHDC1*, and *EIF3H* showed completely opposite changes in expression levels in both aging processes (Fig. 1H). The expression of 5mC regulators *DNMT1*, *IDH2,* and *TET1* were downregulated in both aging stages (Fig. 1I). Transcriptional analysis also revealed that the expression levels of *MMP2* and *CXCL10* increased during both types of senescence, and the mRNA levels of most senescence-associated secretory phenotype (SASP)-related genes were elevated in H_2_O_2_-induced premature senescence and significantly decreased in replicative senescence (Fig. 1J).

### Overview of the m6A methylation map in premature and replicative senescence

To further explore the m6A methylation profiles of replicative senescence and premature senescence, we performed MeRIP-seq analysis. We counted the number of methylation peaks within three replicate experiments for each sample group separately and took the number of intersections of the three replicate experiments.

**Fig 2:**
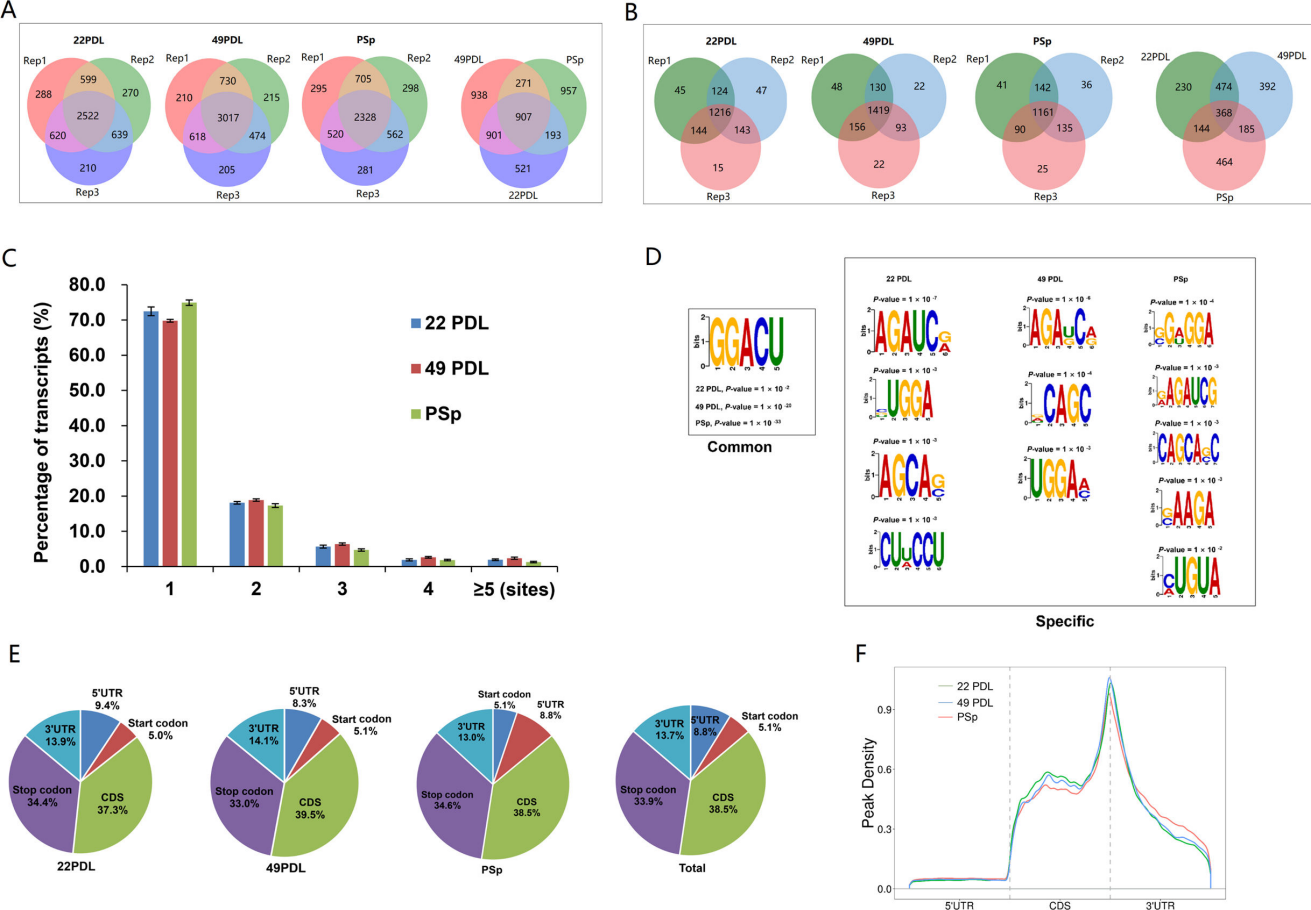
General features of m6A methylation in different groups. (**A**) Venn diagram displaying unique and common m6A peaks within mRNAs in the 22PDL, 49PDL, and PSp groups. (**B**) Venn diagram showing unique and common m6A-modified genes in the 22PDL, 49PDL, and PSp groups. (**C**) The number of genes containing different numbers of m6A peaks and the majority of m6A-modified genes having one to three m6A peaks in the three groups. (**D**) Common and specific motifs enriched from m6A peaks in the three groups separately using HOMER software. (**E**) The proportion of m6A peaks distribution in RNA transcripts in the three groups. The location of m6A peaks in RNA transcripts was divided into 5′ UTR, start codon, coding sequence (CDS), stop codon, and 3′ UTR. (**F**) Density of m6A peaks in RNA transcripts in three groups.

As depicted in Fig. 2A and B, the 22PDL group had 2,522 m6A peaks in 1,216 genes, the 49PDL group had a total of 3,017 m6A peaks within 1,419 genes, and the PSp group had 2,328 m6A peaks within 1,161 genes. Among them, 907 m6A peaks in 368 genes overlapped among the three groups. The 22PDL group had 521 unique m6A peaks and 464 unique m6A-modified genes, the 49PDL group had 938 unique m6A peaks and 230 unique m6A-modified genes, and the PSp group had 957 unique m6A peaks and 392 unique m6A-modified genes. To further analyze the distribution of m6A peaks in each gene, we found that about 70% of m6A-modified genes had only one m6A peak, about 20% had two m6A peaks, and only about 10% had more than three m6A peaks (Fig. 2C). The location of m6A peaks in RNA transcripts was divided into five regions: 5 UTR, start codon, coding sequence (CDS), stop codon, and 3′ UTR. We used HOMER software to analyze the conserved RRACH (R = A/G; H = A/U/C) sequence in m6A peaks. As shown in Fig. 2D, we found nine specific RNA methylation motifs in the 22PDL group, 9 in the 49PDL group, and 12 in the PSp group. The classical m6A motif, GGACU, was present in all three groups, indicating a relative conservation of m6A RNA methylation between H_2_O_2_-induced premature and replicative senescence. As shown in Fig. 2E, we found no significant differences in the distribution sites among the three groups. The combined distribution data of the m6A peaks of the three groups showed that 38.5% of m6A peaks were enriched in the CDS region, followed by 33.9% in the stop codon region, 13.7% in the 3′ UTR region, 8.8% in the 5′ UTR region, and 5.1% in the start codon region. The most common locations of m6A peaks in our study were the CDS, stop codon, and 3′ UTR regions. Furthermore, m6A peaks had the highest density in the stop codon region (Fig. 2F).

### Distribution of differentially methylated m6A peaks and function enrichment and pathway analysis

In comparison to the 22PDL group, the 49PDL group identified a total of 691 differentially hypermethylated m6A peaks within 631 genes and 673 differentially hypomethylated m6A peaks within 608 genes. Meanwhile, the PSp group identified a total of 1042 differentially hypermethylated m6A peaks within 876 genes and 1,460 differentially hypomethylated m6A peaks within 1,106 genes. Table 1 shows the top 10 differentially hypermethylated or hypomethylated m6A peaks for the 49PDL and PSp groups, respectively.

**TABLE 1:** The first 10 differentially hypermethylated or hypomethylated m6A peaks in cell senescence^*a*^

Group	Gene	Log_2_^FC^	*P*	Chr	Peak start	Peak end	Regulation
49PDL vs. 22PDL	BRI3BP	6.007463	<0.0001	chr12	126,000,000	126,000,000	Up
	APOB	108.5	<0.0001	chr2	21,230,241	21,230,740	Up
	KIAA0895L	194.9474	<0.0001	chr16	67,217,164	67,217,480	Up
	ZNF263	75.82911	<0.0001	chr16	3,339,521	3,339,840	Up
	DEPTOR	5.272999	<0.0001	chr8	121,000,000	121,000,000	Up
	PM20D2	433.9	<0.0001	chr6	89,871,827	89,872,180	Up
	IGF1R	12.33962	<0.0001	chr15	99,502,521	99,502,860	Up
	ZNF687	28.31343	<0.0001	chr1	151,000,000	151,000,000	Up
	JPH2	14.96226	<0.0001	chr20	42,744,701	42,745,026	Up
	IGSF10	5.35558	<0.0001	chr3	151,000,000	151,000,000	Up
	SSH1	1,227	<0.0001	chr12	109,000,000	109,000,000	Down
	DUSP2	888.1	<0.0001	chr2	96,809,221	96,809,600	Down
	PCDH1	838.1	<0.0001	chr5	141,000,000	141,000,000	Down
	C1orf198	640.3421	<0.0001	chr1	231,000,000	231,000,000	Down
	TAS2R19	479.0789	<0.0001	chr12	11,174,217	11,174,540	Down
	TRPM7	478.8	<0.0001	chr15	50,849,355	50,849,660	Down
	RBM33	445.3	<0.0001	chr7	156,000,000	156,000,000	Down
	APOL6	436.6	<0.0001	chr22	36,059,801	36,060,180	Down
	PHYHIP	412.3	<0.0001	chr8	22,077,721	22,078,060	Down
	AMPD2	406.2	<0.0001	chr1	110,000,000	110,000,000	Down
PSp vs. 22PDL	SFN	47,862.6	<0.0001	chr1	27,189,632	27,190,947	Up
	CLDN2	22,617.7	<0.0001	chrX	106,000,000	10,6000,000	Up
	ELF3	15,393.8	<0.0001	chr1	202,000,000	202,000,000	Up
	WNT7B	14,046.9	<0.0001	chr22	46,317,901	46,318,720	Up
	KRT81	12,834.16	<0.0001	chr12	52,679,696	52,680,277	Up
	MUC5AC	12,709.3	<0.0001	chr11	1,213,081	1,213,750	Up
	CPLX2	9,486.9	<0.0001	chr5	175,000,000	175,000,000	Up
	AKR1B10	8,917.9	<0.0001	chr7	134,000,000	134,000,000	Up
	MUC5B	7,298.7	<0.0001	chr11	1,268,821	1,269,380	Up
	INHBB	7,279.4	<0.0001	chr2	121,000,000	121,000,000	Up
	PCDH10	103,030	<0.0001	chr4	134,000,000	134,000,000	Down
	CCDC8	76,423.5	<0.0001	chr19	46,915,801	46,916,420	Down
	SULF1	74,277.2	<0.0001	chr8	70,570,739	70,571,780	Down
	ST6GALNAC5	40,598.3	<0.0001	chr1	77,528,659	77,529,640	Down
	FLT1	38,367.6	<0.0001	chr13	28,963,101	28,964,241	Down
	PCDH10	36,197.1	<0.0001	chr4	134,000,000	134,000,000	Down
	COL6A3	28,061	<0.0001	chr2	238,000,000	238,000,000	Down
	KIAA1462	26,071.1	<0.0001	chr10	30,317,841	30,318,795	Down
	LUM	19,928.5	<0.0001	chr12	9,1501,894	91,502,560	Down
	FLRT2	19,182.8	<0.0001	chr14	86,087,482	86,088,920	Down

^*a*^ Up: upregulated genes (Log_2_^FC^ ≥ 2.0, *P* < 0.0001); Down: downregulated genes (Log_2_^FC^ ≤ 0.5, *P* < 0.0001).

**Fig 3:**
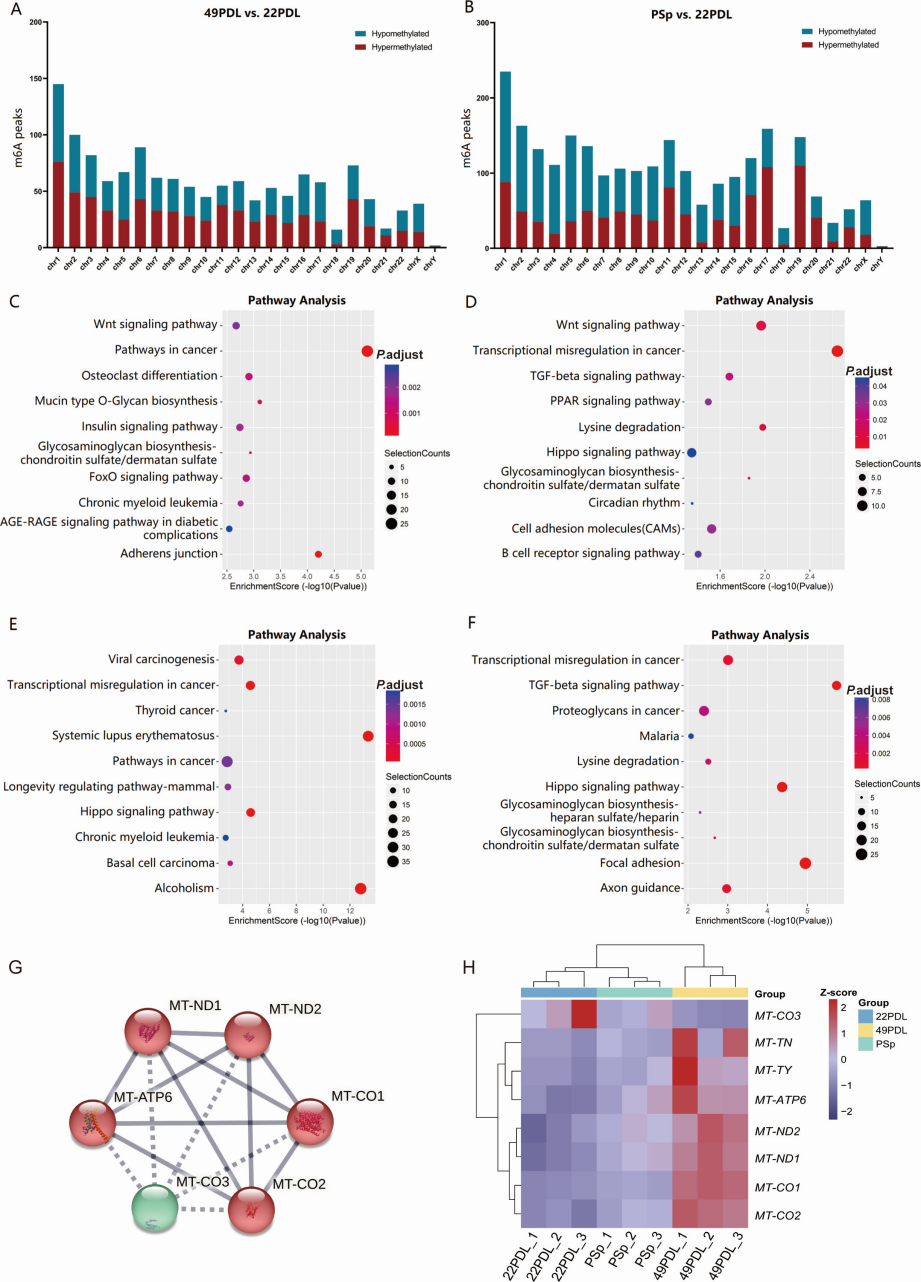
Distribution of differentially methylated m6A peaks and function enrichment and pathway analysis. (**A**) The number of differentially methylated m6A peaks in different chromosomes in replicative senescence. (**B**) The number of differentially methylated m6A peaks in different chromosomes in H _2_O_2_-induced premature senescence. (**C**) KEGG analysis of genes with differentially hypermethylated m6A peaks in replicative senescence. (**D**) KEGG analysis of genes with differentially hypomethylated m6A peaks in replicative senescence. (**E**) KEGG analysis of genes with differentially hypermethylated m6A peaks in H _2_O_2_-induced premature senescence. (**F**) KEGG analysis of genes with differentially hypomethylated m6A peaks in H _2_O_2_-induced premature senescence. (**G**) PPI network of mitochondrial-encoded genes. (**H**) Heatmap of mitochondrial-encoded gene expression across experimental groups.

We further analyzed the distribution of differentially methylated m6A peaks and mapped them to chromosomes. As depicted in Fig. 3A and B, chromosome 1 had the highest number of differentially methylated m6A peaks in both the 49PDL and PSp groups compared to the 22PDL group. In addition, the top five chromosomes with the highest number of differentially hypermethylated m6A peaks in the 49PDL group were chromosomes 1, 2, 6, 5, and 3, and those with the highest number of differentially hypomethylated m6A peaks were chromosomes 1, 2, 6, 19, and 3. In the PSp group, the top five chromosomes with the highest number of differentially hypermethylated m6A peaks were chromosomes 19, 17, 1, 11, and 16, and those with the highest number of differentially hypomethylated m6A peaks were chromosomes 1, 5, 2, 3, and 4.

To elucidate the biological functions and signaling pathways of m6A modification during replicative senescence and H_2_O_2_-induced premature senescence, both GO enrichment and KEGG pathway analyses were carried out. Figure S2 shows the top 10 most significantly enriched Biological processes (BPs), Cellular components (CCs), and Molecular functions (MFs) among the genes up-/down-regulated by m6A peaks in replicative senescence and premature senescence, respectively. Through the KEGG pathway analysis, the crucial pathways of differentially m6A-modified genes were identified. In replicative senescence, genes with differentially hypermethylated m6A peaks were enriched in several key pathways, including pathways in cancer, adherens junction, mucin type O-glycan biosynthesis, Wnt signaling pathway, FoxO signaling pathway, and insulin signaling pathway; while genes with differentially hypomethylated m6A peaks were associated with transcriptional misregulation in cancer, Wnt signaling pathway, lysine degradation, TGF-beta signaling pathway, and cell adhesion molecules. In H_2_O_2_-induced premature senescence, hypermethylated genes were involved in systemic lupus erythematosus, alcoholism, transcriptional misregulation in cancer, the Hippo signaling pathway, and viral carcinogenesis. Hypomethylated genes were involved in the TGF-beta signaling pathway, focal adhesion, Hippo signaling pathway, transcriptional mis-regulation in cancer, and axon guidance (Fig. 3C through F).

From the MeRIP-seq-derived DEGs, we screened the mitochondrial-encoded genes and identified eight with significant alterations, including mitochondrially encoded tRNA-Asn (MT-TN), tRNA-Tyr (MT-TY), ATP synthase membrane subunit 6 (MT-ATP6), NADH: ubiquinone oxidoreductase core subunit (MT-ND1/2), and cytochrome C oxidase subunits 1-3 (MT-CO1/2/3). The PPI analysis revealed robust interactions among these eight mitochondrial-encoded genes. Expression heatmap analysis showed that seven genes (MT-TN, MT-TY, MT-ATP6, MT-ND1, MT-ND2, MT-CO1, and MT-CO2) were progressively upregulated in both PSp and 49PDL groups, whereas MT-CO3 was specifically downregulated in these groups (Fig. 3G and H).

### DO analysis of DEGs of 22PDL-49PDL and 22PDL-PSp in MeRIP-seq

**Fig 4:**
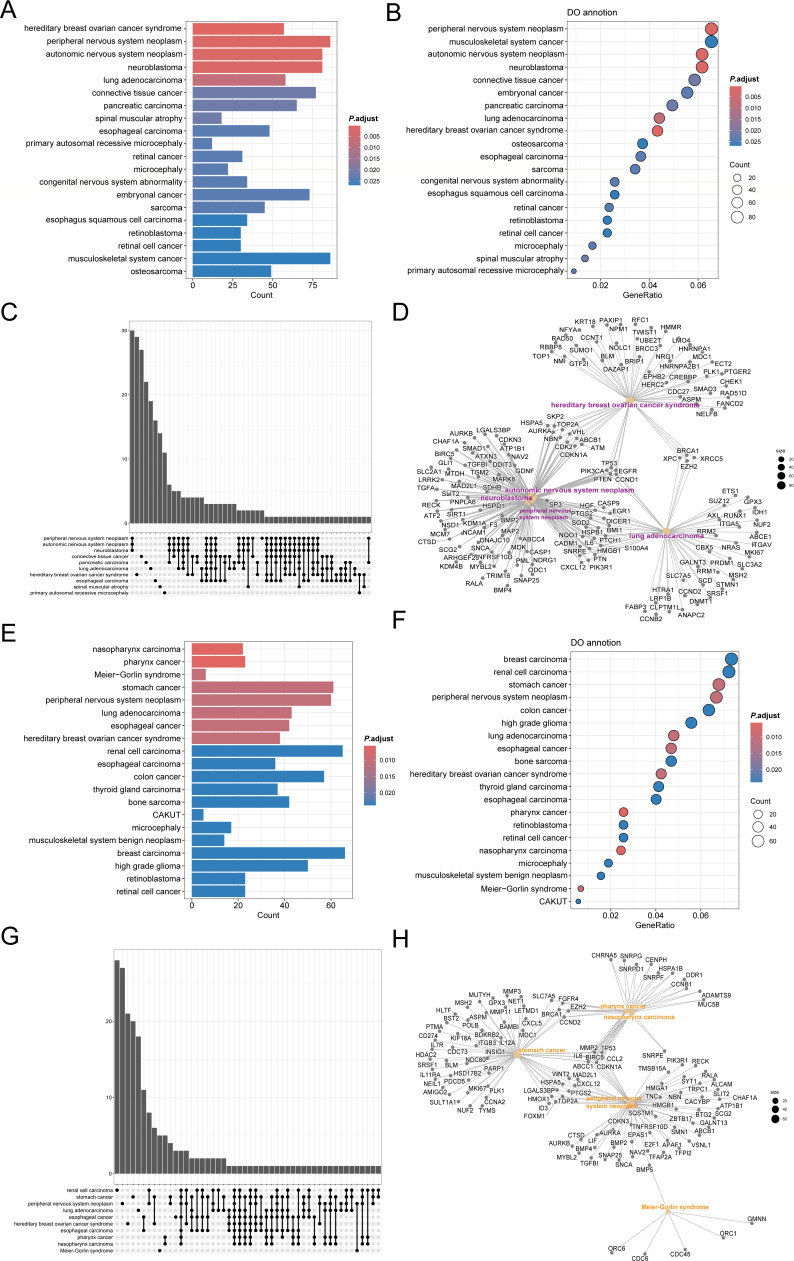
DO analysis of DEGs in MeRIP-seq. (**A, B**) The bar and bubble plots of disease-enriched pathways of DEGs between the 22PDL-49PDL groups. (**C**) The upset maps of overlapping genes between diseases in the 22PDL-49PDL group. (**D**) The network plot of overlapping genes between the 22PDL-49PDL groups. (**E, F**) The bar and bubble plots of disease-enriched pathways of DEGs between the 22PDL-PSp groups. (**G**) The upset maps of overlapping genes between diseases in the 22PDL-PSp group. (**H**) The network plot of overlapping genes between the 22PDL-PSp groups.

To further explore the similarities and differences in the degree of m6A methylation in replicative and premature senescence at the disease level, we conducted an enrichment analysis of DEGs in MeRIP-seq using DO analysis for 22PDL with 49PDL and PSp groups, respectively. According to the bar and bubble plots (Fig. 4A, B, E and F), replicative senescence and premature senescence showed consistent enrichment in hereditary breast ovarian cancer syndrome, peripheral nervous system neoplasm, lung adenocarcinoma, esophageal carcinoma, retinal cancer, retinoblastoma, retinal cell cancer, microcephaly, and osteosarcoma diseases. It was notable that DEGs of 22PDL-49PDL were more associated with highly malignant cancers such as connective tissue cancer, pancreatic carcinoma, esophagus squamous cell carcinoma, and musculoskeletal system cancer. While the DEGs of 22PDL-PSp were closely associated with nasopharyngeal carcinoma, pharyngeal cancer, stomach cancer, renal cell carcinoma, colon cancer, thyroid gland carcinoma, breast carcinoma, and musculoskeletal system benign neoplasm. Figure 4C, D, G, and H shows that there is multiple synergistic regulation of the same genes between the enriched disorders and the diseases. The DEGs of 22PDL-49PDL were the most enriched in overlapping genes in peripheral nervous system neoplasm, autonomic nervous system neoplasm, and neuroblastoma, whereas the DEGs for 22PDL-PSp were most overlapped in stomach cancer, peripheral nervous system neoplasm, pharynx cancer, and nasopharynx carcinoma.

### Global DNA methylation patterns in premature and replicative senescence

**Fig 5:**
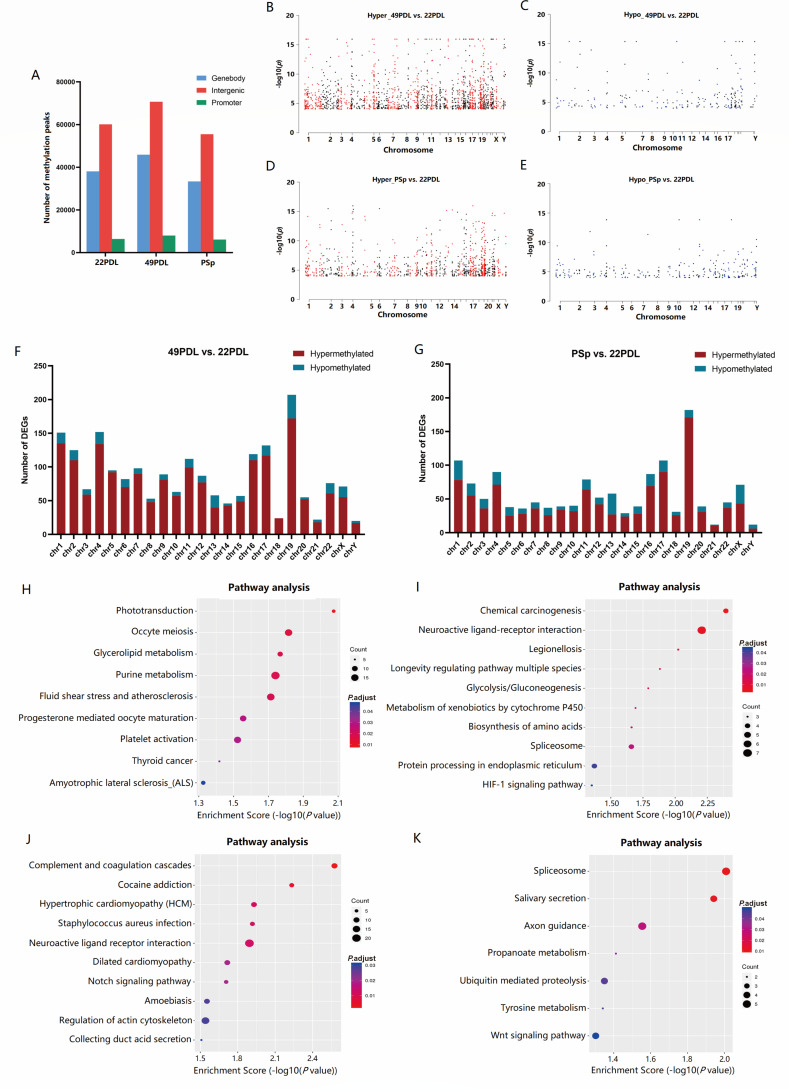
Distribution of differentially methylated 5mC peaks and function enrichment and pathway analysis. (**A**) The proportion of 5mC peaks distribution in three groups. Manhattan plots of DMRs on chromosomes showed that replicative senescence exhibited (**B**) 1,810 hypermethylated and (**C**) 251 hypomethylated DMRs; H_2_O_2_-induced premature senescence exhibited (**D**) 1,090 hypermethylated and (**E**) 308 hypomethylated genes. (**F**) The number of differentially methylated 5mC peaks in different chromosomes in replicative senescence. (**G**) The number of differentially methylated 5mC peaks in different chromosomes in H_2_O_2_-induced premature senescence. (**H**) KEGG analysis of genes with differentially hypermethylated m6A peaks in replicative senescence. (**I**) KEGG analysis of genes with differentially hypermethylated 5mC peaks in replicative senescence. (**J**) KEGG analysis of genes with differentially hypermethylated 5mC peaks in H_2_O_2_-induced premature senescence. (**K**) KEGG analysis of genes with differentially hypomethylated 5mC peaks in H_2_O_2_-induced premature senescence.

Similarly, we conducted an analysis of global DNA methylation between different senescent cells using MeDIP-seq. In the 22PDL group, we identified 104,593 methylation peaks, in the 49PDL group 124,535 methylation peaks, and in the PSp group 94,943 methylation peaks. We then calculated the proportion of methylation peaks in different functional elements, revealing that methylation peaks were primarily distributed in intergenic regions and gene bodies (Fig. 5A). Compared to the 22PDL group, 2061 differentially methylated genes were found in the 49PDL group, of which 1,810 (87.82%) were hypermethylated and 251 (12.18%) were hypomethylated; while 1,398 differentially methylated genes were found in the PSp group, of which 1,090 (77.97%) were hypermethylated and 308 (22.03%) were hypomethylated. Manhattan plots (Fig. 5B through E) illustrate the overall distribution of DNA methylation in H_2_O_2_-induced premature and replicative senescence. Then, the distribution of differentially methylated peaks was analyzed and mapped to chromosomes. As shown in Fig. 5F and G, chromosome 19 had the highest number of differentially methylated peaks in both the 49PDL and PSp groups compared to the 22PDL group.

To elucidate the biological functions and signaling pathways of DNA 5mC modifications during replicative and H_2_O_2_-induced premature senescence, we performed GO enrichment and KEGG pathway analysis. Figure S3 lists the top 10 most significantly enriched BPs, CCs, and MFs among genes with up/downregulation of 5mC peaks in replicative senescence and premature senescence. KEGG pathway analysis (Fig. 4H through K) identified key pathways of genes with different 5mC modifications. Genes with different levels of hypermethylation in replicative senescence were enriched in several key pathways, including phototransduction, oocyte meiosis, glycerolipid metabolism, purine metabolism and fluid shear stress, and atherosclerosis. Genes with different hypomethylated 5mC peaks were associated with chemical carcinogenesis, neuroactive ligand-receptor interaction, longevity regulation pathway multiple species, and the spliceosome. In H_2_O_2_-induced premature senescence, hypermethylated genes were involved in complement and coagulation cascades, neuroactive ligand receptor interaction, and regulation of actin cytoskeleton; the hypomethylated genes were involved in the spliceosome, salivary secretion, axon guidance, and Wnt signaling pathway.

### DO analysis of DEGs of 22PDL-49PDL and 22PDL-PSp in MeDIP-seq

**Fig 6:**
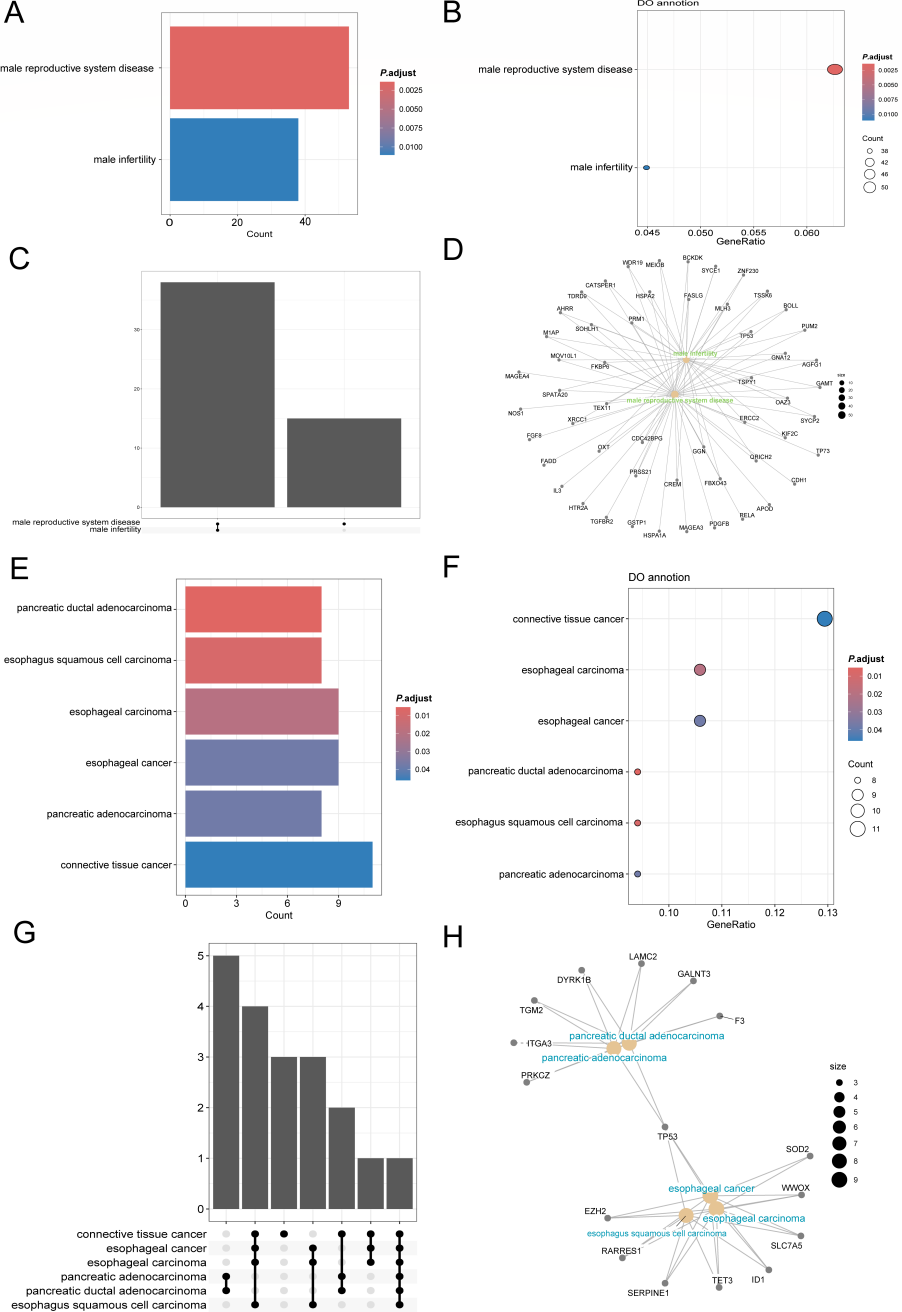
DO analysis of DEGs in MeDIP-seq. (**A, B**) The bar and bubble plots of disease-enriched pathways of DEGs between the 22PDL-49PDL groups. (**C**) The upset maps of overlapping genes between diseases in the 22PDL-49PDL group. (**D**) The network plot of overlapping genes between the 22PDL-49PDL groups. (**E, F**) The bar and bubble plots of disease-enriched pathways of DEGs between the 22PDL-PSp groups. (**G**) The upset maps of overlapping genes between diseases in the 22PDL-PSp group. (**H**) The network plot of overlapping genes between the 22PDL-PSp groups.

Likewise, we utilized MeDIP-seq of DEGs in 22PDL-49PDL and 22PDL-PSp to conduct DO enrichment analysis. It was shown that DEGs of 22PDL-49PDL were enriched in male reproductive system disease and male infertility diseases and were co-regulated by multiple genes (Fig. 6A through D). Compared to 22PDL-49PDL, 22PDL-PSp had more DEGs-enriched diseases, including pancreatic ductal adenocarcinoma, esophagus squamous cell carcinoma, esophageal carcinoma, pancreatic adenocarcinoma, and connective tissue cancer (Fig. 6E and F). Based on Fig. 6G and H, there were five co-genes enriched in pancreatic ductal adenocarcinoma and pancreatic adenocarcinoma disease. And the esophageal cancer, esophageal carcinoma, and esophagus squamous cell carcinoma also shared common regulatory genes. Moreover, there was a common overlapping gene, TP53, in all five of these diseases.

### Interaction between RNA m6A and DNA 5mC in premature and replicative senescence

**Fig 7:**
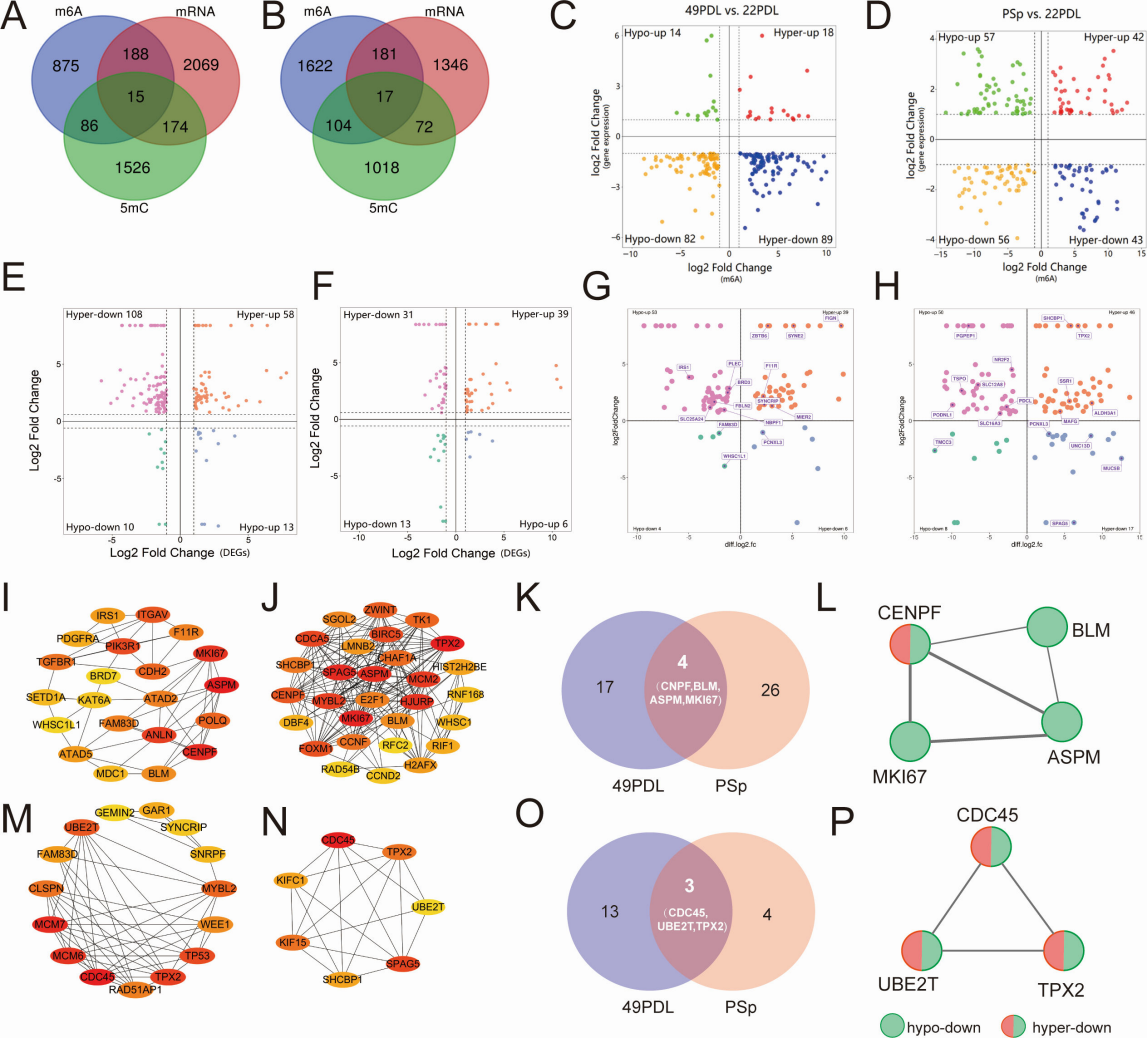
Crosstalk between RNA m6A and DNA 5mC in premature and replicative senescence. (**A, B**) Venn diagrams of DEGs, m6A methylated genes, and 5mC methylated differential genes in the 49PDL and PSp groups. (**C, D**) Four-quadrant graphs showed the DEGs with differentially methylated m6A peaks in the 49PDL and PSp groups, separately. (**E, F**) Four-quadrant graphs showed the DEGs with differentially methylated 5mC peaks in the 49PDL and PSp groups, separately. (**G, H**) Four-quadrant graphs showed the differentially methylated m6A genes with differentially methylated 5mC peaks in the 49PDL and PSp groups, separately. (**I, J**) A total of 20 hub genes were identified from 203 genes in the 49PDL, and a total of 30 hub genes were identified from 198 genes in the PSp group by MCC algorithm analysis. Network nodes represent proteins; edges represent protein-protein associations. Red was the higher score calculated by the MCC method, followed by yellow. (**K**) Venn diagram showing the four common hub genes of the 49PDL and PSp groups. (**L**) PPI network was constructed by four common hub genes of the 49PDL and PSp groups. Wider lines indicated stronger evidence of protein interactions. (**M, N**) A total of 15 hub genes were identified from 189 genes in the 49PDL, and a total of 7 hub genes were identified from 89 genes in the PSp group by the MCC algorithm analysis. (**O**) Venn diagram showing the three common hub genes of the 49PDL and PSp groups. (**P**) PPI network was constructed by three common hub genes of the 49PDL and PSp groups.

Based on the integrated analysis of MeRIP-seq and RNA-seq data, genes showing significant changes in both m6A modification and RNA expression levels in the 49PDL and PSp groups were identified (Fig. 7A and B). Differentially expressed genes with differentially methylated m6A peaks were categorized into four groups. In replicative senescence and H_2_O_2_-induced premature senescence, 203 and 198 DEGs with differential m6A peaks were identified, respectively. The four-quadrant plots in Fig. 7C and D suggested a potential correlation between differentially methylated m6A peaks and gene expression levels. Specifically, 189 and 89 DEGs with differential m6A peaks were identified in the 49PDL and PSp groups, respectively (Fig. 7E and F). All these genes were listed in Tables S1 and S2.

To further explore the relationship between these DEGs and their m6A modifications, we constructed a protein-protein interaction network using the STRING database and visualized it with Cytoscape software. Hub genes were then screened. As shown in Fig. 7I and J, 21 hub genes were identified in the 49PDL group, while 30 hub genes were identified in the PSp group, compared to the 22PDL group. The hub genes for each group were listed in Table 2. Common hub genes were identified to uncover shared pathways and targets in both the 49PDL and PSp groups. The results revealed that the common pivotal genes in both groups were abnormal spindle-like microcephaly-associated (*ASPM*), centromere protein F (*CENPF*), marker of proliferation Ki-67 (*MKI67*), and Bloom helicase (*BLM*), all of which are closely associated with mitosis and cell cycle regulation (Fig. 5K). As shown in Fig. 7L, strong interactions were observed among the proteins encoded by these four genes. In both the 49PDL and PSp groups, *ASPM*, *MKI67*, and *BLM* were downregulated genes with hypomethylated m6A peaks (hypo-down), while *CENPF* was a downregulated gene with hypermethylated m6A peaks. Western blot analysis of these four hub genes showed that the expression level of *BLM* significantly increased, while *CENPF* significantly decreased in both the 49PDL and PSp groups. In addition, the expression levels of *ASPM* and *MKi67* were significantly elevated in the PSp group (Fig. 5Q).

**TABLE 2:** The hub genes of the 49PDL and PSp groups obtained by MCC screening^*a*^

Group	Gene	Score	Gene expression			m6A		
			Log_2_^FC^	*P*	Regulation	Log_2_^FC^	*P*	Regulation
49PDL vs. 22PDL	ASPM	88	−4.58708	<0.0001	Down	−1.77163	<0.0001	Down
	CENPF	84	−2.86829	0.00065	Down	3.794578	<0.0001	Up
	MKI67	81	−3.55421	<0.0001	Down	−1.66764	<0.0001	Down
	ANLN	79	−3.11061	0.00005	Down	−4.06534	<0.0001	Down
	PIK3R1	43	−2.57005	0.0008	Down	4.505379	<0.0001	Up
	ITGAV	32	−1.07455	<0.0001	Down	1.809838	<0.0001	Up
	CDH2	30	−1.74943	<0.0001	Down	−1.24006	<0.0001	Down
	POLQ	30	−3.53631	0.03135	Down	3.472006	<0.0001	Up
	TGFBR1	29	−1.01885	0.00015	Down	−1.62363	<0.0001	Down
	F11R	26	3.55486	0.00635	Up	2.197205	<0.0001	Up
	FAM83D	26	−2.2765	0.0391	Down	−2.06679	<0.0001	Down
	ATAD2	26	−3.22549	0.00005	Down	2.592007	<0.0001	Up
	BLM	19	−4.40138	<0.0001	Down	−3.49244	<0.0001	Down
	IRS1	16	−1.78678	<0.0001	Down	−4.96935	<0.0001	Down
	ATAD5	16	−2.10678	0.0099	Down	−5.37009	<0.0001	Down
	PDGFRA	15	−1.08218	<0.0001	Down	1.54969	<0.0001	Up
	SETD1A	14	−1.07047	0.00965	Down	2.110483	<0.0001	Up
	MUDC1	14	−2.21301	<0.0001	Down	−2.27368	<0.0001	Down
	KAT6A	13	−1.39347	<0.0001	Down	−7.56758	<0.0001	Down
	WHSC1L1	8	−1.67115	<0.0001	Down	−1.54658	<0.0001	Down
	BRD7	8	−1.20445	0.02205	Down	5.215894	<0.0001	Up
PSp vs. 22PDL	TPX2	490000000	−2.80357	<0.0001	Down	6.786431	<0.0001	Up
	MKI67	490000000	−2.94977	0.00115	Down	6.086693	<0.0001	Up
	SPAG5	490000000	−2.95754	0.01685	Down	6.246192	<0.0001	Up
	ASPM	490000000	−3.93102	<0.0001	Down	−3.58803	<0.0001	Down
	HJURP	490000000	−3.89466	<0.0001	Down	6.373223	<0.0001	Up
	MCM2	486000000	−1.23313	0.0235	Down	4.973251	<0.0001	Up
	MYBL2	486000000	−3.68576	0.0021	Down	8.157487	<0.0001	Up
	CDCA5	486000000	−3.55158	<0.0001	Down	8.484219	<0.0001	Up
	BIRC5	486000000	−3.64644	<0.0001	Down	8.260397	<0.0001	Up
	FOXM1	486000000	−3.10803	0.0097	Down	6.377154	<0.0001	Up
	CENPF	486000000	−2.37006	<0.0001	Down	2.914599	<0.0001	Up
	ZWINT	486000000	−2.07402	0.00315	Down	7.350939	<0.0001	Up
	TK1	483000000	−2.5321	<0.0001	Down	8.559271	<0.0001	Up
	CHAF1A	3675913	−1.82985	0.0192	Down	4.605293	<0.0001	Up
	CCNF	3674907	−2.47682	<0.0001	Down	8.531254	<0.0001	Up
	SHCBP1	3669120	−3.79383	<0.0001	Down	5.823273	<0.0001	Up
	E2F1	51388	−1.20815	0.0224	Down	7.538423	<0.0001	Up
	SGOL2	40345	−2.01358	<0.0001	Down	−4.74444	<0.0001	Down
	BLM	1558	−3.33685	0.0041	Down	−8.20457	<0.0001	Down
	LMNB2	756	−1.13272	0.03375	Down	5.752489	<0.0001	Up
	H2AFX	679	−1.04373	0.0239	Down	4.268295	<0.0001	Up
	HIST2H2BE	118	2.40939	<0.0001	Up	4.854794	<0.0001	Up
	RIF1	96	−1.07656	0.0019	Down	−6.30695	<0.0001	Down
	DBF4	73	−1.65855	0.0052	Down	−2.64698	<0.0001	Down
	WHSC1	48	−1.41997	0.0071	Down	4.845048	<0.0001	Up
	RNF168	33	−1.3087	0.0071	Down	−3.08855	<0.0001	Down
	CCND2	26	3.72775	<0.0001	Up	−9.64926	<0.0001	Down
	RAD54B	14	−2.83747	0.0066	Down	−9.07708	<0.0001	Down
	RFC2	10	−1.24724	0.0156	Down	11.15634	<0.0001	Up
	HIST1H1C	8	1.94654	0.00085	Up	3.529158	<0.0001	Up

^*a*^ Up: upregulated genes (Log_2_^FC^ ≥ 1, *P* < 0.0001); Down: downregulated genes (Log_2_^FC^ ≤ −1, *P* < 0.001).

Furthermore, the top five hub genes specific to the 49PDL group were Anillin (*ANLN*), phosphoinositide-3-kinase regulatory subunit 1 (*PIK3R1*), integrin subunit alpha V (*ITGAV*), cadherin 2 (*CDH2*), and DNA polymerase theta (*POLQ*). By contrast, the top five hub genes unique to the PSp group were TPX2 microtubule nucleation factor (*TPX2*), sperm-associated antigen 5 (*SPAG5*), Holliday junction recognition protein (*HJURP*), minichromosome maintenance complex component 2 (*MCM2*), and MYB proto-oncogene like 2 (*MYBL2*). These results suggest that genes with m6A modifications play a critical role in cellular senescence, with *ASPM*, *CENPF*, *MKI67*, and *BLM* potentially serving as common target genes for m6A modifications in both replicative and premature senescence groups.

As shown in Fig. 7M and N, compared to the 22PDL group, 15 hub genes were identified in the 49PDL group, while 7 hub genes were identified in the PSp group. The hub genes for each group are listed in Table 3. Common hub genes were identified to uncover shared pathways and targets in both groups. The results revealed that the common pivotal genes in both groups were *CDC45*, *TPX2*, and *UBE2T*, all of which were closely associated with mitosis and cell cycle regulation (Fig. 7O). As shown in Fig. 7P, strong interactions were observed among the proteins encoded by these three genes. In addition, the top four hub genes exclusive to the 49PDL group were *MCM7*, *MCM6*, *TP53*, and *MYBL2*, while the top four hub genes unique to the PSp group were *SPAG5*, *KIF15*, *SHCBP1*, and *KIFC1*. These results indicated that genes with 5mC modifications significantly contributed to cellular senescence, with *CDC45*, *TPX2*, and *UBE2T* potentially serving as common target genes for 5mC modifications in both replicative and premature senescence groups.

**TABLE 3:** The hub genes of the 49PDL and PSp groups obtained by MCC screening^*a*^

Group	Gene	Score	Gene expression			m6A		
			Log_2_^FC^	*P*	Regulation	Log_2_^FC^	*P*	Regulation
49PDL vs. 22PDL	CDC45	1250	−4.63574	<0.0001	Down	2.00	0.003185	Up
	MCM7	1201	−2.26444	<0.0001	Down	3.11	0.014951	Up
	MCM6	1200	−1.96575	<0.0001	Down	3.09	0.000815	Up
	TPX2	1131	−3.3078	<0.0001	Down	8.38	0.000652	Up
	TP53	990	−3.41048	<0.0001	Down	2.03	0.000346	Up
	UBE2T	891	−2.6419	0.00135	Down	3.71	0.0001	Up
	MYBL2	745	−3.70409	0.02175	Down	8.38	0.000401	Up
	CLSPN	362	−4.29435	<0.0001	Down	8.38	0.004388	Up
	RAD51AP1	264	−4.10785	<0.0001	Down	3.17	0.010443	Up
	WEE1	124	−1.49206	0.00185	Down	2.17	0.011369	Up
	FAM83D	48	−2.2765	0.0391	Down	−1.10	0.020215	Down
	GAR1	28	−1.3190	0.00075	Down	4.83	<0.0001	Up
	SNRPF	27	−1.81876	0.00005	Down	8.38	<0.0001	Up
	SYNCRIP	25	−1.57112	0.00665	Down	1.42	<0.0001	Up
	GEMIN2	24	−1.69137	0.0184	Down	1.61	<0.0001	Up
PSp vs. 22PDL	CDC45	147	−3.19596	0.0014	Down	2.15	<0.0001	Up
	SPAG5	145	−2.95754	0.01685	Down	−8.99	<0.0001	Down
	TPX2	144	−2.80357	0.00005	Down	8.38	<0.0001	Up
	KIF15	144	−2.62324	0.00145	Down	−3.32	<0.0001	Down
	SHCBP1	120	−3.79383	0.00005	Down	8.38	<0.0001	Up
	KIFC1	120	−2.64791	0.01825	Down	3.39	<0.0001	Up
	UBE2T	26	−2.02194	0.0003	Down	3.64	<0.0001	Up

^*a*^ Up: upregulated genes (Log_2_^FC^ ≥ 1, *P* < 0.0001); Down: downregulated genes (Log_2_^FC^ ≤ −1, *P* < 0.001).

To further identify genes regulated by both DNA 5mC and RNA m6A methylation during different senescence processes, we performed an integrated analysis of DEGs, m6A differentially methylated genes, and 5mC differentially methylated genes. In the replicative senescence group, 377 DEGs were regulated by either one or both modifications, with 15 genes regulated by both methylation modifications. In the H_2_O_2_-induced premature senescence group, 269 DEGs were regulated by either one or both modifications, with 17 genes regulated by both methylation modifications (Fig. 7A and B). As shown in Fig. 7G and H, in replicative senescence, 43 genes exhibited a positive correlation between changes in RNA m6A and DNA 5mC methylation levels, while 59 genes showed a negative correlation. In H_2_O_2_-induced premature senescence, 56 genes displayed a positive correlation between changes in RNA m6A and DNA 5mC methylation levels, while 67 genes exhibited a negative correlation.

These findings collectively highlighted the intricate interplay between RNA m6A and DNA 5mC modifications in regulating cellular senescence, with key genes such as *ASPM*, *CENPF*, *MKI67*, *BLM*, *CDC45*, *TPX2*, and *UBE2T* playing pivotal roles in these processes.

### Correlation and expression analysis of hub genes for RNA m6A and DNA 5mC

**Fig 8:**
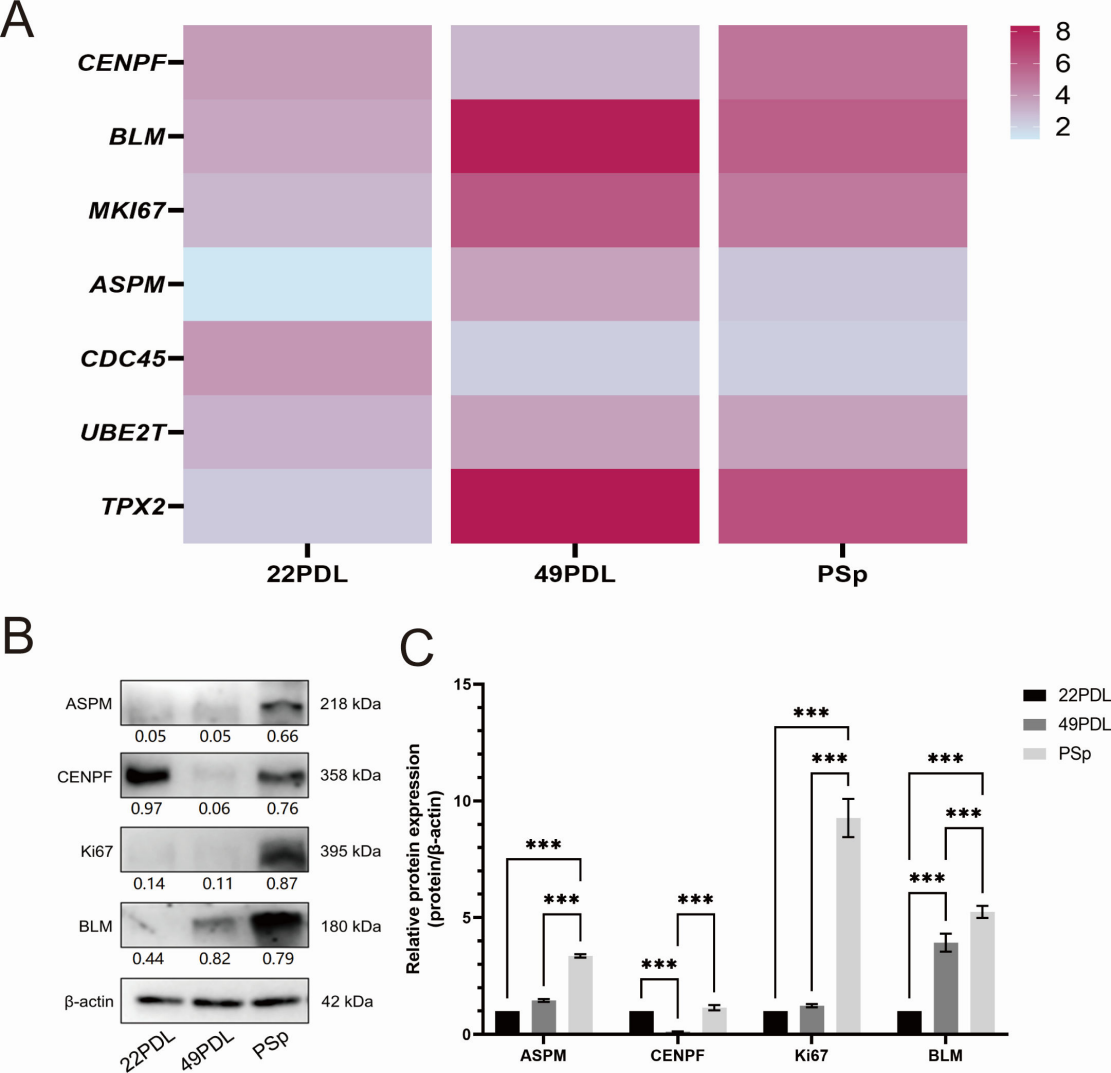
Correlation analysis of target genes and validation by Western blot. (**A**) Heatmap of expression correlation of target genes in sequencing results across groups. (**B**) Western blot analysis for ASPM, CENPF, Ki67, and BLM in 22PDL, 49PDL, and PSp groups. (**C**) Quantitative analysis of the Western blot results (***P < 0.001).

To further investigate the expression patterns of hub genes across different groups, we first performed a correlation analysis on the expression levels of 7 hub genes in each group based on sequencing data. The heatmap results revealed that, compared to the 22PDL group, the expression of hub genes was generally elevated in the 49PDL and PSp groups. Western blot results showed that, compared to the 22PDL group, the protein expression levels of ASPM and BLM were increased in the 49PDL group, while the expression levels of ASPM, CENPF, Ki67, and BLM were significantly elevated in the PSp group. Furthermore, compared to the 49PDL group, the protein expression levels of ASPM, CENPF, Ki67, and BLM were even more markedly increased in the PSp group (Fig. 8A through C). These results suggested that the proteins ASPM, CENPF, Ki67, and BLM, associated with RNA m6A, played crucial regulatory roles in the cellular senescence process.

## DISCUSSION

HEFs were frequently and widely used for cellular senescence research, with H_2_O_2_ serving as a model environmental pollutant inducing premature cellular senescence. The molecular mechanisms underlying H_2_O_2_-induced premature and replicative cellular senescence and the global patterns of m6A and 5mC modifications in HEFs remain incompletely understood. While the m6A modifications exhibit dynamic reversibility under stress, their regulatory roles in senescence require further investigation (32). DNA methylation patterns vary during senescence, with localized hypermethylation occurring at heterochromatin foci, though overall methylation levels remain stable in stress-induced premature senescence (33, 34). Our results provided the first comprehensive overview of RNA m6A and DNA 5mC modifications during replicative and H_2_O_2_-induced premature senescence, revealing their collaborative role in senescence regulation.

Through MeRIP-seq and MeDIP-seq analyses, we observed distinct modification patterns between senescence models. In comparison to replicative senescence, the premature senescence group exhibited an increase in 5mC methylation genes and modification sites, while m6A methylation genes and modification sites decreased. This suggested that the inherent cause of H_2_O_2_-induced premature aging may be the increase in the number of 5mC-methylated genes suppressing the transcription of relevant genes, whereas the decrease in the number of m6A-methylated genes within the cells mediates the regulation of gene expression at the post-transcriptional level, thereby altering post-transcriptional function. Consistent with cancer, the distribution of m6A peaks in H_2_O_2_-induced premature and replicative senescent cells was predominantly in the CDS (35). Large amounts of m6A modifications in the CDS regulate RNA stability, transport, and translation (36). Therefore, we hypothesized that when cells enter the proliferative and migratory state, changes in genetic and microenvironmental factors would be mainly concentrated at the beginning and end of the CDS. It is worth noting that the common motif of the three groups of cells is GGACU, which suggests that although m6A is widely present on mRNA, and its modification site is relatively conserved.

The GO and KEGG enrichment analysis of genes with varying degrees of methylation of 5mC and m6A peaks revealed that the two modifications were involved in distinct biological processes and pathways in replicative senescence and premature senescence cells. In replicative senescence, KEGG results indicated that genes differentially methylated in the 5mC peak were mainly concentrated in the glycerol ester metabolism and longevity regulatory pathways. Disruption of *in vivo* lipid homeostasis underlies age-related diseases, and glyceryl triacetate attenuates hippocampal neuronal senescence and improves cognitive function in aged rats (37, 38). By contrast, m6A-modified genes predominantly participated in oncogenic signaling pathways, including the TGF-β cascade, which promotes ROS generation and senescent cell accumulation in pulmonary fibrosis (39, 40). In H_2_O_2_-induced premature aging cells, genes differentially methylated at the 5mC peak were mainly enriched in the complement and coagulation cascades and actin cytoskeleton regulation, which can activate the pro-inflammatory properties of cells *in vivo* to affect cellular senescence and disease progression (41, 42). Meanwhile, genes differentially methylated at the m6A peak are mainly enriched in focal adhesion and cancer-related signaling pathways. Mechanistically, impaired focal adhesion in senescent cells reduces nitric oxide bioavailability, triggering inflammatory cascades that reinforce senescence (41). In both types of senescent cells, 5mC modification and m6A modification have different potential functions, suggesting that different epigenetic modifications may regulate the process of cellular senescence through diverse pathways and functions.

RNA m6A methylation and its regulators were strongly associated with several diseases, including non-alcoholic fatty liver disease, heart failure, and cancer (43). Similarly, DNA 5mC serves as both an epigenetic biomarker and functional regulator, with its dysregulation implicated in carcinogenesis, neurodegenerative diseases, and Rett syndrome (44). We used DO analysis to project disease enrichment of RNA m6A and DNA 5mC in replicative aging and premature aging. Our findings are consistent with previous studies that both types of epigenetics are highly correlated with cancer. Using DO analysis of 22PDL-49PDL and 22PDL-PSp DEGs in MeRIP-seq, compared to premature senescence, more DEGs in replicative senescence were strongly associated with malignant tumors such as pancreatic cancer, esophageal squamous cell carcinoma, etc. The m6A-modified homeostatic imbalances could affect cancer development by modulating the signaling pathways of key tumor suppressors and oncogenes. Mechanistically, m6A imbalance promotes oncogenesis by altering tumor suppressor and oncogene signaling—exemplified by METTL14-driven m6A modifications accelerating pancreatic cancer metastasis, and viral-induced METTL3 overexpression exacerbating esophageal carcinoma progression (45, 46). In MeDIP-seq, gene expression in prematurely senescent cells was more enriched in malignant tumors compared to replicative senescence. DO analysis revealed the similarities and differences between different cellular senescence states and diseases at the epigenetic level, which provided a new reference for subsequent research on clinical therapeutic targets and preventive interventions against senescence.

Using the STRING database, we assessed 15 and 7 hub genes regulated by 5mC modification in replicative senescent and premature senescent cells, respectively. Subsequently, in the replicative senescent and premature senescent groups, we identified three core genes involved in mitosis and cell cycle regulation: CDC45, TPX2, and UBE2T. CDC45 is a component of the eukaryotic DNA replication helicase significantly decreased in chronically quiescent, terminally differentiated, and senescent human cells (47). TPX2 is a microtubule-associated protein discovered in the nucleus of S-phase and G2-phase and can be involved in the regulation of mitotic spindle architecture, apoptotic events, mitosis, cytokinesis, and cell proliferation (48, 49). Targeting TPX2 further impedes cell cycle progression, amplifies genomic instability, and accelerates cellular senescence (50). Moreover, mouse embryonic fibroblasts lacking TPX2 eventually exit mitosis without chromosome segregation (51). Through increased activation of checkpoint kinase 1 (CHK1), UBE2T induces a DNA damage response and boosts G2/M arrest (52). UBE2T is a critical E2 enzyme involved in the anemia pathway’s DNA repair, and its mediated ubiquitination regulates multiple cancer-related pathways (53). The silencing of UBE2T leads to an increase in the proportion of cells in G2/M phase and a decrease in the proportion of cells in G1 phase, resulting in cell cycle arrest in the G2/M phase (54).

Similarly, we screened 21 and 30 hub genes regulated by m6A modification in replicative senescent and premature aging cells, respectively. Through common pathways and targets in the replicative senescent and premature aging groups, we pinpointed four key genes involved in cell cycle regulation: *ASPM*, *CENPF*, *MKI67*, and *BLM*. *ASPM*, a novel classical Wnt-β-catenin signaling co-regulator, participates in spindle organization, spindle localization, and cytoplasmic division across all dividing cells (55, 56). The isoform ASPM-iII, predominantly localized in the nucleus, interacts with the cell cycle protein E to mainly regulate the G1/S transition (57). METTL3 upregulates CEP170 expression through m6A modification, thereby activating downstream ASPM expression. This METTL3-m6A-CEP170-ASPM regulatory axis not only promotes esophageal cancer cell proliferation and maintains spindle stability but also leads to chromosome segregation errors and microtubule instability, when dysfunctional, ultimately accelerating cellular senescence via genomic instability pathways (58). CENPF, mainly involved in the mitotic process of cell cycle chromosome segregation, is expressed in a cell cycle-associated pattern, peaking in the G2/M phase of the cell cycle (59). Previous studies have demonstrated that downregulation of the m6A reader protein LRPPRC promotes abnormal proliferation of pulmonary arterial smooth muscle cells through relieving transcriptional suppression of CENPF. Dysregulation of the LRPPRC-m6A-CENPF regulatory axis could accelerate cellular senescence via genomic instability pathways, linking to the cancer-like pathological mechanisms observed in pulmonary arterial hypertension (60). METTL3-mediated m6A modification enhances CENPF mRNA stability via HNRNPA2B1 recognition, thereby activating the senescence-associated MAPK signaling pathway and promoting FAK cytoplasmic localization. Moreover, its high expression is significantly associated with lymph node infiltration and poor patient prognosis (61). *MKI67* reflects the proliferative phase of the cell, and the level of its encoded protein, Ki-67, can be assessed in the nucleus of cells in G1, S, and G2 phases and mitotic divisions (62). MKI67 functions as a critical m6A methylation-regulated hub target, mediating inflammation associated with intracranial aneurysm (63). *BLM*, an ATP-dependent DNA deconjugating enzyme, plays a key role in several steps of the DNA recombination, replication, and repair process, ensuring chromosome segregation in human cells (64, 65). METTL3-mediated m6A modification increases the expression of lncRNA PVT1. This LncRNA sequesters miR-27b-3p, thereby alleviating its suppression on BLM, which, in turn, promotes the proliferative, migratory, and invasive capacities of prostate cancer cells (66). All four hub genes are closely associated with various cancers, and their m6A modification levels align with their gene expression levels, suggesting that changes in m6A modification levels may influence the expression of the hub genes and thus regulate the associated diseases (67).

Currently, small-molecule inhibitors targeting m6A modification (including STM2457, 3-Deazaadenosine) and DNA demethylating agents (including azacitidine, decitabine) have entered clinical application, with azacitidine and decitabine having received FDA approval for the treatment of myelodysplastic syndromes (68–70). Through systematic screening, this study has successfully identified three key target genes regulated by 5mC methylation and four key target genes regulated by m6A methylation. These findings provide an important foundation for developing novel gene therapy strategies. However, current epigenetic therapies still face two major challenges: the targeting specificity of existing modulators needs improvement, and the complexity of epigenetic regulatory networks increases therapeutic difficulty. The seven epigenetically regulated target genes identified in this study could offer new insights into disease mechanisms related to cellular senescence and establish a molecular basis for developing targeted therapeutic strategies.

This study integrated multi-omics analyses (MeRIP-seq and MeDIP-seq) to elucidate the epigenetic mechanisms by which H_2_O_2_ coordinately drives cellular senescence, especially through the regulation of both DNA 5mC and RNA m6A methylation modifications. We systematically demonstrated a complete molecular cascade linking environmental oxidative stress, the epigenetic microenvironment, and the senescence phenotype. Furthermore, we successfully identified hub senescence-regulating downstream genes characterized by both 5mC and m6A modification signatures. These genes not only serve as novel epigenetic biomarkers of aging but also provide a theoretical foundation for the development of anti-aging therapies targeting coordinated DNA and RNA methylation regulation. Importantly, our findings demonstrate that transcriptional-level (5mC) and post-transcriptional-level (m6A) epigenetic modifications exhibit marked, important synergistic effects in regulating senescence-associated pathways, including oxidative stress response and DNA damage repair. The discovery of this cross-layer epigenetic regulatory network offers profound mechanistic insights into cellular senescence.

### Conclusion

In summary, this study was the first to demonstrate both roles of RNA m6A methylation and DNA 5mC methylation on a transcriptomic scale in replicative and premature senescent cells. We found that the same motifs of m6A modification were present in two cellular senescence groups, and most of the biological processes were focused on cancer-related signaling pathways, including the TGF-β signaling pathway, Wnt signaling pathway, and transcriptional mis-regulation in cancer. However, compared to the replicative senescence group, the premature senescence group exhibited an increase in 5mC methylation modification sites and a decrease in m6A methylation modification sites. Importantly, we discovered that *ASPM*, *CENPF*, *MKI67*, and *BLM* were the most frequently targeted genes for m6A modification, and *CDC45*, *TPX2*, and *UBE2T* were the most frequently targeted genes for 5mC modification in the replicative and premature senescence groups.

## Data Availability

The authors state that the main data supporting the results of this study are presented in the paper. Additional data are available on request from the corresponding authors. The data sets presented in this study can be found in online repositories. The names of repositories and accession websites can be found in the article.

## SUPPLEMENTAL MATERIAL

Supplemental material msystems.01647-24-s0001.docx Tables S1 and S2; Figures S1 to S3.

ASM does not own the copyrights to Supplemental Material that may be linked to, or accessed through, an article. The authors have granted ASM a non-exclusive, world-wide license to publish the Supplemental Material files. Please contact the corresponding author directly for reuse.

## References

[1] Campisi J, Kapahi P, Lithgow GJ, Melov S, Newman JC, Verdin E. 2019. From discoveries in ageing research to therapeutics for healthy ageing. Nature 571:183–192. doi:10.1038/s41586-019-1365-2 31292558 PMC7205183

[2] Aghali A, Koloko Ngassie ML, Pabelick CM, Prakash YS. 2022. Cellular senescence in aging lungs and diseases. Cells 11:1781. doi:10.3390/cells11111781 35681476 PMC9179897

[3] López-Otín C, Blasco MA, Partridge L, Serrano M, Kroemer G. 2023. Hallmarks of aging: an expanding universe. Cell 186:243–278. doi:10.1016/j.cell.2022.11.001 36599349

[4] Gorgoulis V, Adams PD, Alimonti A, Bennett DC, Bischof O, Bishop C, Campisi J, Collado M, Evangelou K, Ferbeyre G, et al.. 2019. Cellular senescence: defining a path forward. Cell 179:813–827. doi:10.1016/j.cell.2019.10.005 31675495

[5] Barnes PJ, Baker J, Donnelly LE. 2019. Cellular senescence as a mechanism and target in chronic lung diseases. Am J Respir Crit Care Med 200:556–564. doi:10.1164/rccm.201810-1975TR 30860857

[6] Egger G, Liang G, Aparicio A, Jones PA. 2004. Epigenetics in human disease and prospects for epigenetic therapy. Nature 429:457–463. doi:10.1038/nature02625 15164071

[7] Zhu X, Chen Z, Shen W, Huang G, Sedivy JM, Wang H, Ju Z. 2021. Inflammation, epigenetics, and metabolism converge to cell senescence and ageing: the regulation and intervention. Signal Transduct Target Ther 6:245. doi:10.1038/s41392-021-00646-9 34176928 PMC8236488

[8] Zhang H, Sun D, Li D, Zheng Z, Xu J, Liang X, Zhang C, Wang S, Wang J, Lu W. 2018. Long non-coding RNA expression patterns in lung tissues of chronic cigarette smoke induced COPD mouse model. Sci Rep 8:7609. doi:10.1038/s41598-018-25702-3 29765063 PMC5954018

[9] Schübeler D. 2015. Function and information content of DNA methylation. Nature 517:321–326. doi:10.1038/nature14192 25592537

[10] Issa JP, Ahuja N, Toyota M, Bronner MP, Brentnall TA. 2001. Accelerated age-related CpG island methylation in ulcerative colitis. Cancer Res 61:3573–3577. 11325821

[11] Lund G, Andersson L, Lauria M, Lindholm M, Fraga MF, Villar-Garea A, Ballestar E, Esteller M, Zaina S. 2004. DNA methylation polymorphisms precede any histological sign of atherosclerosis in mice lacking apolipoprotein E. J Biol Chem 279:29147–29154. doi:10.1074/jbc.M403618200 15131116

[12] Dayeh T, Volkov P, Salö S, Hall E, Nilsson E, Olsson AH, Kirkpatrick CL, Wollheim CB, Eliasson L, Rönn T, Bacos K, Ling C. 2014. Genome-wide DNA methylation analysis of human pancreatic islets from type 2 diabetic and non-diabetic donors identifies candidate genes that influence insulin secretion. PLoS Genet 10:e1004160. doi:10.1371/journal.pgen.1004160 24603685 PMC3945174

[13] Tohgi H, Utsugisawa K, Nagane Y, Yoshimura M, Genda Y, Ukitsu M. 1999. Reduction with age in methylcytosine in the promoter region −224∼−101 of the amyloid precursor protein gene in autopsy human cortex. Mol Brain Res 70:288–292. doi:10.1016/S0169-328X(99)00163-1 10407177

[14] Lewinska A, Adamczyk-Grochala J, Kwasniewicz E, Deregowska A, Semik E, Zabek T, Wnuk M. 2018. Reduced levels of methyltransferase DNMT2 sensitize human fibroblasts to oxidative stress and DNA damage that is accompanied by changes in proliferation-related miRNA expression. Redox Biol 14:20–34. doi:10.1016/j.redox.2017.08.012 28843151 PMC5568885

[15] Xie W, Kagiampakis I, Pan L, Zhang YW, Murphy L, Tao Y, Kong X, Kang B, Xia L, Carvalho FLF, Sen S, Chiu Yen R-W, Zahnow CA, Ahuja N, Baylin SB, Easwaran H. 2018. DNA methylation patterns separate senescence from transformation potential and indicate cancer risk. Cancer Cell 33:309–321. doi:10.1016/j.ccell.2018.01.008 29438699 PMC5813821

[16] Wu F, Zhang L, Lai C, Peng X, Yu S, Zhou C, Zhang B, Zhang W. 2022. Dynamic alteration profile and new role of RNA m6A methylation in replicative and H_2_O_2_-induced premature senescence of human embryonic lung fibroblasts. Int J Mol Sci 23:9271. doi:10.3390/ijms23169271 36012545 PMC9408987

[17] Chen X, Yu C, Guo M, Zheng X, Ali S, Huang H, Zhang L, Wang S, Huang Y, Qie S, Wang J. 2019. Down-regulation of m6A mRNA methylation is involved in dopaminergic neuronal death. ACS Chem Neurosci 10:2355–2363. doi:10.1021/acschemneuro.8b00657 30835997

[18] Tang C, Xie Y, Yu T, Liu N, Wang Z, Woolsey RJ, Tang Y, Zhang X, Qin W, Zhang Y, Song G, Zheng W, Wang J, Chen W, Wei X, Xie Z, Klukovich R, Zheng H, Quilici DR, Yan W. 2020. m6A-dependent biogenesis of circular RNAs in male germ cells. Cell Res 30:211–228. doi:10.1038/s41422-020-0279-8 32047269 PMC7054367

[19] Zaccara S, Ries RJ, Jaffrey SR. 2019. Reading, writing and erasing mRNA methylation. Nat Rev Mol Cell Biol 20:608–624. doi:10.1038/s41580-019-0168-5 31520073

[20] Wu Z, Shi Y, Lu M, Song M, Yu Z, Wang J, Wang S, Ren J, Yang Y-G, Liu G-H, Zhang W, Ci W, Qu J. 2020. METTL3 counteracts premature aging via m6A-dependent stabilization of MIS12 mRNA. Nucleic Acids Res 48:11083–11096. doi:10.1093/nar/gkaa816 33035345 PMC7641765

[21] Min K-W, Zealy RW, Davila S, Fomin M, Cummings JC, Makowsky D, Mcdowell CH, Thigpen H, Hafner M, Kwon S-H, Georgescu C, Wren JD, Yoon J-H. 2018. Profiling of m6A RNA modifications identified an age-associated regulation of AGO2 mRNA stability. Aging Cell 17:e12753. doi:10.1111/acel.12753 29573145 PMC5946072

[22] Fan T, Du Y, Zhang M, Zhu AR, Zhang J. 2022. Senolytics cocktail dasatinib and quercetin alleviate human umbilical vein endothelial cell senescence via the TRAF6-MAPK-NF-κB Axis in a YTHDF2-dependent manner. Gerontology 68:920–934. doi:10.1159/000522656 35468611

[23] Tian Y, Xiao H, Yang Y, Zhang P, Yuan J, Zhang W, Chen L, Fan Y, Zhang J, Cheng H, Deng T, Yang L, Wang W, Chen G, Wang P, Gong P, Niu X, Zhang X. 2023. Crosstalk between 5-methylcytosine and N^6^-methyladenosine machinery defines disease progression, therapeutic response and pharmacogenomic landscape in hepatocellular carcinoma. Mol Cancer 22:5. doi:10.1186/s12943-022-01706-6 36627693 PMC9830866

[24] Yang X, Mei C, Raza SHA, Ma X, Wang J, Du J, Zan L. 2022. Interactive regulation of DNA demethylase gene TET1 and m6A methyltransferase gene METTL3 in myoblast differentiation. Int J Biol Macromol 223:916–930. doi:10.1016/j.ijbiomac.2022.11.081 36375665

[25] Chien WW, Ffrench M. 2006. Régulation de p16INK4a, senescence et oncogenèse. Médecine/Sciences 22:865–871. doi:10.1051/medsci/20062210865 17026941

[26] Zhang L, Wan Y, Zhang Z, Jiang Y, Gu Z, Ma X, Nie S, Yang J, Lang J, Cheng W, Zhu L. 2021. IGF2BP1 overexpression stabilizes PEG10 mRNA in an m6A-dependent manner and promotes endometrial cancer progression. Theranostics 11:1100–1114. doi:10.7150/thno.49345 33391523 PMC7738899

[27] Wang Q, Wang W, Zhang A. 2021. TET-mediated DNA demethylation plays an important role in arsenic-induced HBE cells oxidative stress via regulating promoter methylation of OGG1 and GSTP1. Toxicol In Vitro 72:105075. doi:10.1016/j.tiv.2020.105075 33388378

[28] Lin L, Hu X, Li Q, Huang L. 2024. Methyltransferase-like 3 (METTL3) epigenetically modulates glutathione peroxidase 4 (GPX4) expression to affect asthma. Iran J Allergy Asthma Immunol 22:551–560. doi:10.18502/ijaai.v22i6.14644 38477952

[29] Campisi J. 1997. The biology of replicative senescence. Eur J Cancer 33:703–709. doi:10.1016/S0959-8049(96)00058-5 9282108

[30] Kural KC, Tandon N, Skoblov M, Kel-Margoulis OV, Baranova AV. 2016. Pathways of aging: comparative analysis of gene signatures in replicative senescence and stress induced premature senescence. BMC Genomics 17:1030. doi:10.1186/s12864-016-3352-4 28105936 PMC5249001

[31] Wang Y, Gao J, Wu F, Lai C, Li Y, Zhang G, Peng X, Yu S, Yang J, Wang W, Zhang W, Yang X. 2021. Biological and epigenetic alterations of mitochondria involved in cellular replicative and hydrogen peroxide-induced premature senescence of human embryonic lung fibroblasts. Ecotoxicol Environ Saf 216:112204. doi:10.1016/j.ecoenv.2021.112204 33845364

[32] He PC, He C. 2021. m6A RNA methylation: from mechanisms to therapeutic potential. EMBO J 40:e105977. doi:10.15252/embj.2020105977 33470439 PMC7849164

[33] Narita M, Nũnez S, Heard E, Narita M, Lin AW, Hearn SA, Spector DL, Hannon GJ, Lowe SW. 2003. Rb-mediated heterochromatin formation and silencing of E2F target genes during cellular senescence. Cell 113:703–716. doi:10.1016/s0092-8674(03)00401-x 12809602

[34] Koch CM, Reck K, Shao K, Lin Q, Joussen S, Ziegler P, Walenda G, Drescher W, Opalka B, May T, Brümmendorf T, Zenke M, Saric T, Wagner W. 2013. Pluripotent stem cells escape from senescence-associated DNA methylation changes. Genome Res 23:248–259. doi:10.1101/gr.141945.112 23080539 PMC3561866

[35] Zhang Z, Wang Q, Zhang M, Zhang W, Zhao L, Yang C, Wang B, Jiang K, Ye Y, Shen Z, Wang S. 2021. Comprehensive analysis of the transcriptome-wide m6A methylome in colorectal cancer by MeRIP sequencing. Epigenetics 16:425–435. doi:10.1080/15592294.2020.1805684 32749190 PMC7993153

[36] Batista PJ, Molinie B, Wang J, Qu K, Zhang J, Li L, Bouley DM, Lujan E, Haddad B, Daneshvar K, Carter AC, Flynn RA, Zhou C, Lim K-S, Dedon P, Wernig M, Mullen AC, Xing Y, Giallourakis CC, Chang HY. 2014. m^6^A RNA modification controls cell fate transition in mammalian embryonic stem cells. Cell Stem Cell 15:707–719. doi:10.1016/j.stem.2014.09.019 25456834 PMC4278749

[37] Gille B, Galuska CE, Fuchs B, Peleg S. 2021. Recent advances in studying age-associated lipids alterations and dietary interventions in mammals. Front Aging 2:773795. doi:10.3389/fragi.2021.773795 35822042 PMC9261446

[38] Lv S, Zhang Y, Lin Y, Fang W, Wang Y, Li Z, Lin A, Dai X, Ye Q, Zhang J, Chen X. 2023. ApoE4 exacerbates the senescence of hippocampal neurons and spatial cognitive impairment by downregulating acetyl-CoA level. Aging Cell 22:e13932. doi:10.1111/acel.13932 37594184 PMC10497817

[39] Yoon Y-S, Lee J-H, Hwang S-C, Choi KS, Yoon G. 2005. TGF β1 induces prolonged mitochondrial ROS generation through decreased complex IV activity with senescent arrest in Mv1Lu cells. Oncogene 24:1895–1903. doi:10.1038/sj.onc.1208262 15688038

[40] Minagawa S, Araya J, Numata T, Nojiri S, Hara H, Yumino Y, Kawaishi M, Odaka M, Morikawa T, Nishimura SL, Nakayama K, Kuwano K. 2011. Accelerated epithelial cell senescence in IPF and the inhibitory role of SIRT6 in TGF-β-induced senescence of human bronchial epithelial cells. Am J Physiol Lung Cell Mol Physiol 300:L391–L401. doi:10.1152/ajplung.00097.2010 21224216 PMC3284316

[41] Moiseeva V, Cisneros A, Sica V, Deryagin O, Lai Y, Jung S, Andrés E, An J, Segalés J, Ortet L, Lukesova V, Volpe G, Benguria A, Dopazo A, Benitah SA, Urano Y, Del Sol A, Esteban MA, Ohkawa Y, Serrano AL, Perdiguero E, Muñoz-Cánoves P. 2023. Senescence atlas reveals an aged-like inflamed niche that blunts muscle regeneration. Nature 613:169–178. doi:10.1038/s41586-022-05535-x 36544018 PMC9812788

[42] Iram T, Kern F, Kaur A, Myneni S, Morningstar AR, Shin H, Garcia MA, Yerra L, Palovics R, Yang AC, Hahn O, Lu N, Shuken SR, Haney MS, Lehallier B, Iyer M, Luo J, Zetterberg H, Keller A, Zuchero JB, Wyss-Coray T. 2022. Young CSF restores oligodendrogenesis and memory in aged mice via Fgf17. Nature 605:509–515. doi:10.1038/s41586-022-04722-0 35545674 PMC9377328

[43] Jiang X, Liu B, Nie Z, Duan L, Xiong Q, Jin Z, Yang C, Chen Y. 2021. The role of m6A modification in the biological functions and diseases. Signal Transduct Target Ther 6:74. doi:10.1038/s41392-020-00450-x 33611339 PMC7897327

[44] Wang J, Tang J, Lai M, Zhang H. 2014. 5-Hydroxymethylcytosine and disease. Mutat Res Rev Mutat Res 762:167–175. doi:10.1016/j.mrrev.2014.09.003 25475423

[45] Chen S, Yang C, Wang Z-W, Hu J-F, Pan J-J, Liao C-Y, Zhang J-Q, Chen J-Z, Huang Y, Huang L, Zhan Q, Tian Y-F, Shen B-Y, Wang Y-D. 2021. CLK1/SRSF5 pathway induces aberrant exon skipping of METTL14 and Cyclin L2 and promotes growth and metastasis of pancreatic cancer. J Hematol Oncol 14:60. doi:10.1186/s13045-021-01072-8 33849617 PMC8045197

[46] Guo S, Chen F, Li L, Dou S, Li Q, Huang Y, Li Z, Liu W, Zhang G. 2024. Intracellular Fusobacterium nucleatum infection increases METTL3-mediated m6A methylation to promote the metastasis of esophageal squamous cell carcinoma. J Adv Res 61:165–178. doi:10.1016/j.jare.2023.08.014 37619934 PMC11258656

[47] Pollok S, Bauerschmidt C, Sänger J, Nasheuer H-P, Grosse F. 2007. Human Cdc45 is a proliferation-associated antigen. FEBS J 274:3669–3684. doi:10.1111/j.1742-4658.2007.05900.x 17608804

[48] Chang H, Wang J, Tian Y, Xu J, Gou X, Cheng J. 2012. The TPX2 gene is a promising diagnostic and therapeutic target for cervical cancer. Oncol Rep 27:1353–1359. doi:10.3892/or.2012.1668 22307108

[49] Zhang L, Huang H, Deng L, Chu M, Xu L, Fu J, Zhu Y, Zhang X, Liu S, Zhou Z, Wang Y. 2008. TPX2 in malignantly transformed human bronchial epithelial cells by anti-benzo[a]pyrene-7,8-diol-9,10-epoxide. Toxicology 252:49–55. doi:10.1016/j.tox.2008.07.059 18723071

[50] Chen Y-C, Chen I-S, Huang G-J, Kang C-H, Wang K-C, Tsao M-J, Pan H-W. 2018. Targeting DTL induces cell cycle arrest and senescence and suppresses cell growth and colony formation through TPX2 inhibition in human hepatocellular carcinoma cells. Onco Targets Ther 11:1601–1616. doi:10.2147/OTT.S147453 29606879 PMC5868578

[51] Aguirre-Portolés C, Bird AW, Hyman A, Cañamero M, Pérez de Castro I, Malumbres M. 2012. Tpx2 controls spindle integrity, genome stability, and tumor development. Cancer Res 72:1518–1528. doi:10.1158/0008-5472.CAN-11-1971 22266221

[52] Sun J, Zhu Z, Li W, Shen M, Cao C, Sun Q, Guo Z, Liu L, Wu D. 2020. UBE2T-regulated H2AX monoubiquitination induces hepatocellular carcinoma radioresistance by facilitating CHK1 activation. J Exp Clin Cancer Res 39:222. doi:10.1186/s13046-020-01734-4 33087136 PMC7576867

[53] Dutta R, Guruvaiah P, Reddi KK, Bugide S, Reddy Bandi DS, Edwards YJK, Singh K, Gupta R. 2022. UBE2T promotes breast cancer tumor growth by suppressing DNA replication stress. NAR Cancer 4:zcac035. doi:10.1093/narcan/zcac035 36338541 PMC9629447

[54] Liu L-L, Zhu J-M, Yu X-N, Zhu H-R, Shi X, Bilegsaikhan E, Guo H-Y, Wu J, Shen X-Z. 2019. UBE2T promotes proliferation via G2/M checkpoint in hepatocellular carcinoma. Cancer Manag Res 11:8359–8370. doi:10.2147/CMAR.S202631 31571992 PMC6750879

[55] Hsu C-C, Liao W-Y, Chan T-S, Chen W-Y, Lee C-T, Shan Y-S, Huang P-J, Hou Y-C, Li C-R, Tsai KK. 2019. The differential distributions of ASPM isoforms and their roles in Wnt signaling, cell cycle progression, and pancreatic cancer prognosis. J Pathol 249:498–508. doi:10.1002/path.5341 31465125 PMC6899738

[56] Jiang K, Rezabkova L, Hua S, Liu Q, Capitani G, Altelaar AFM, Heck AJR, Kammerer RA, Steinmetz MO, Akhmanova A. 2017. Microtubule minus-end regulation at spindle poles by an ASPM–katanin complex. Nat Cell Biol 19:480–492. doi:10.1038/ncb3511 28436967 PMC5458804

[57] Hsu C, Liao W, Chan T, Chen W, Lee C, Shan Y, Huang P, Hou Y, Li C, Tsai KK. 2019. The differential distributions of ASPM isoforms and their roles in Wnt signaling, cell cycle progression, and pancreatic cancer prognosis. J Pathol 249:498–508. doi:10.1002/path.5341 31465125 PMC6899738

[58] Ren K, Luan Y, Yang Y, Xia C, Zhao X, Yan D, He H, Jue B, Yin F, Wu K, Zhang X, Qin B. 2025. METTL3-mediated CEP170 m6A modifications in spindle orientation and esophageal cancer cell proliferation. Int Immunopharmacol 146:113780. doi:10.1016/j.intimp.2024.113780 39708485

[59] Fritzler MJ, Rattner JB, Luft LM, Edworthy SM, Casiano CA, Peebles C, Mahler M. 2011. Historical perspectives on the discovery and elucidation of autoantibodies to centromere proteins (CENP) and the emerging importance of antibodies to CENP-Ftibodies to CENP-F. Autoimmun Rev 10:194–200. doi:10.1016/j.autrev.2010.09.025 20933614

[60] Feng Y, Yu Z, Tang M, Li J, Peng B, Juaiti M, Tang Y, Liang B, Ouyang M, Liu Q, Song J. 2024. Transcriptome-wide N6-methyladenosine alternations in pulmonary arteries of monocrotaline-induced pulmonary arterial hypertension in rats and novel therapeutic targets. Biomedicines 12:364. doi:10.3390/biomedicines12020364 38397966 PMC10886831

[61] Xu P, Yang J, Chen Z, Zhang X, Xia Y, Wang S, Wang W, Xu Z. 2023. N6-methyladenosine modification of CENPF mRNA facilitates gastric cancer metastasis via regulating FAK nuclear export. Cancer Commun (Lond) 43:685–705. doi:10.1002/cac2.12443 37256823 PMC10259669

[62] Wu S-Y, Liao P, Yan L-Y, Zhao Q-Y, Xie Z-Y, Dong J, Sun H-T. 2021. Correlation of MKI67 with prognosis, immune infiltration, and T cell exhaustion in hepatocellular carcinoma. BMC Gastroenterol 21:416. doi:10.1186/s12876-021-01984-2 34724892 PMC8561917

[63] Li S, Zhang Q, Weng L, Han Y, Li J. 2022. Novel insight into m6A regulator-mediated methylation modification patterns and immune characteristics in intracranial aneurysm. Front Aging Neurosci 14:973258. doi:10.3389/fnagi.2022.973258 36034129 PMC9404377

[64] Booth DG, Takagi M, Sanchez-Pulido L, Petfalski E, Vargiu G, Samejima K, Imamoto N, Ponting CP, Tollervey D, Earnshaw WC, Vagnarelli P. 2014. Ki-67 is a PP1-interacting protein that organises the mitotic chromosome periphery. Elife 3:e01641. doi:10.7554/eLife.01641 24867636 PMC4032110

[65] Cuylen S, Blaukopf C, Politi AZ, Müller-Reichert T, Neumann B, Poser I, Ellenberg J, Hyman AA, Gerlich DW. 2016. Ki-67 acts as a biological surfactant to disperse mitotic chromosomes. Nature 535:308–312. doi:10.1038/nature18610 27362226 PMC4947524

[66] Chen B, Liu C, Long H, Bai G, Zhu Y, Xu H. 2023. N^6^-methyladenosine-induced long non-coding RNA PVT1 regulates the miR-27b-3p/BLM axis to promote prostate cancer progression. Int J Oncol 62:16. doi:10.3892/ijo.2022.5464 36484368 PMC9747193

[67] Wen K, Zhang Y, Li Y, Wang Q, Sun J. 2021. Comprehensive analysis of transcriptome-wide m^6^A methylome in the anterior capsule of the lens of high myopia patients. Epigenetics 16:955–968. doi:10.1080/15592294.2020.1834917 33108260 PMC8451460

[68] Yankova E, Blackaby W, Albertella M, Rak J, De Braekeleer E, Tsagkogeorga G, Pilka ES, Aspris D, Leggate D, Hendrick AG, Webster NA, Andrews B, Fosbeary R, Guest P, Irigoyen N, Eleftheriou M, Gozdecka M, Dias JML, Bannister AJ, Vick B, Jeremias I, Vassiliou GS, Rausch O, Tzelepis K, Kouzarides T. 2021. Small-molecule inhibition of METTL3 as a strategy against myeloid leukaemia. Nature 593:597–601. doi:10.1038/s41586-021-03536-w 33902106 PMC7613134

[69] Wang X, Zhu H, Sun G, Zhou M, Zhang H, Liu H, Wang M, Zhang Z, Chu H. 2023. linc01515 regulates PM2.5-induced oxidative stress via targeting NRF2 in airway epithelial cells. Environ Pollut 331:121798. doi:10.1016/j.envpol.2023.121798 37169236

[70] Poloni A, Goteri G, Zizzi A, Serrani F, Trappolini S, Costantini B, Mariani M, Olivieri A, Catarini M, Centurioni R, Alesiani F, Giantomassi F, Stramazzotti D, Biagetti S, Alfonsi S, Berardinelli E, Leoni P. 2013. Prognostic role of immunohistochemical analysis of 5 mc in myelodysplastic syndromes. Eur J Haematol 91:219–227. doi:10.1111/ejh.12145 23679560

