# Supplementary material for: Global m6A RNA and whole 5mC DNA methylation specifically contribute to cell replicative and premature senescence induced by extrinsic oxidative stress
Source: mSystems. 2025 Jun 16;10(7):e01647-24. doi: 10.1128/msystems.01647-24 (PMC12327851; doi:10.1128/msystems.01647-24)
Supplement: Supplemental material — Tables S1 and S2; Figures S1 to S3. [file msystems.01647-24-s0001.docx]

Supplemental Table 1. The 203 differentially expressed genes with different m6A peaks in the 49PDL group.

| Gene | Gene expression | | | m6A | | |
| --- | --- | --- | --- | --- | --- | --- |
|  | Log_2_^FC^ | *P* | Regulation | Log_2_^FC^ | *P* | Regulation |
| ADAMTS8 | -3.36326 | 0.003 | down | 2.846008 | <0.0001 | up |
| AHNAK2 | 1.01062 | 0.00215 | up | 6.620361 | <0.0001 | up |
| AIM1 | -3.33628 | 0.001 | down | 5.226102 | <0.0001 | up |
| AMIGO2 | -1.24454 | 0.0045 | down | -1.68402 | <0.0001 | down |
| ANGPTL4 | 5.71998 | <0.0001 | up | -2.32909 | <0.0001 | down |
| ANKRD12 | -1.16695 | 0.01775 | down | -2.46582 | <0.0001 | down |
| ANKRD50 | -1.02662 | 0.00015 | down | 5.462853 | <0.0001 | up |
| ANLN | -3.11061 | <0.0001 | down | -4.06534 | <0.0001 | down |
| AP3M2 | -1.29233 | 0.03735 | down | -5.13699 | <0.0001 | down |
| APOL6 | -1.32402 | <0.0001 | down | -8.77017 | <0.0001 | down |
| APOLD1 | -2.23304 | 0.00455 | down | 2.393965 | <0.0001 | up |
| ARHGAP12 | -1.01424 | 0.01625 | down | -1.93267 | <0.0001 | down |
| ARID2 | -2.25851 | 0.0118 | down | 4.512921 | <0.0001 | up |
| ASNSD1 | -1.7367 | <0.0001 | down | -2.43225 | <0.0001 | down |
| ASPM | -4.58708 | <0.0001 | down | -1.77163 | <0.0001 | down |
| ASXL2 | -1.371 | <0.0001 | down | 4.842185 | <0.0001 | up |
| ATAD2 | -3.22549 | <0.0001 | down | 2.592007 | <0.0001 | up |
| ATAD5 | -2.10678 | 0.0099 | down | -5.37009 | <0.0001 | down |
| ATL3 | -1.63595 | 0.00255 | down | 7.998308 | <0.0001 | up |
| ATP2B4 | 1.48943 | <0.0001 | up | -2.15404 | <0.0001 | down |
| AZI2 | -1.06092 | 0.0492 | down | -2.7375 | <0.0001 | down |
| B4GALT5 | -1.11965 | <0.0001 | down | 2.915663 | <0.0001 | up |
| BBX | -1.19967 | 0.00525 | down | -2.63185 | <0.0001 | down |
| BLM | -4.40138 | <0.0001 | down | -3.49244 | <0.0001 | down |
| BMP4 | -2.07558 | 0.0037 | down | 4.506589 | <0.0001 | up |
| BRD3 | -1.10904 | 0.0028 | down | -1.23917 | <0.0001 | down |
| BRD7 | -1.20445 | 0.02205 | down | 5.215894 | <0.0001 | up |
| BRI3BP | -1.59935 | 0.0035 | down | 2.586756 | <0.0001 | up |
| BTBD11 | -2.53733 | 0.00285 | down | 9.471269 | <0.0001 | up |
| BTBD7 | -1.94961 | 0.0166 | down | 2.588734 | <0.0001 | up |
| C17orf51 | -1.26175 | 0.004 | down | 4.527326 | <0.0001 | up |
| C1orf131 | -1.71494 | <0.0001 | down | 3.500138 | <0.0001 | up |
| C9orf72 | -1.83736 | 0.037 | down | 5.029218 | <0.0001 | up |
| CARD11 | -inf | 0.04255 | up | -2.77435 | <0.0001 | down |
| CCDC34 | -2.57026 | <0.0001 | down | 2.136039 | <0.0001 | up |
| CCDC8 | -1.82454 | 0.02145 | down | 3.243578 | <0.0001 | up |
| CCDC82 | -1.28693 | <0.0001 | down | -1.93397 | <0.0001 | down |
| CCNT1 | -1.05349 | 0.00015 | down | -2.70037 | <0.0001 | down |
| CCP110 | -1.92279 | 0.0325 | down | 3.003217 | <0.0001 | up |
| CDH2 | -1.74943 | <0.0001 | down | -1.24006 | <0.0001 | down |
| CDK19 | -1.32519 | 0.00025 | down | -2.38091 | <0.0001 | down |
| CDV3 | -1.01011 | 0.00015 | down | 1.153973 | <0.0001 | up |
| CEBPZ | -1.21456 | 0.04495 | down | 1.752327 | <0.0001 | up |
| CENPF | -2.86829 | 0.00065 | down | 3.794578 | <0.0001 | up |
| CHAF1A | -1.96305 | 0.01065 | down | -2.31382 | <0.0001 | down |
| CHD7 | -5.43659 | <0.0001 | down | 1.624945 | <0.0001 | up |
| CHML | -1.07694 | 0.01015 | down | 4.077255 | <0.0001 | up |
| CHSY3 | -1.90294 | 0.03365 | down | -1.25806 | <0.0001 | down |
| COL6A3 | -1.98649 | <0.0001 | down | -2.34119 | <0.0001 | down |
| CRISPLD2 | -2.03487 | 0.00105 | down | 6.788321 | <0.0001 | up |
| CSGALNACT2 | -1.13202 | <0.0001 | down | 6.265829 | <0.0001 | up |
| CSNK1G3 | -1.58529 | <0.0001 | down | -1.47786 | <0.0001 | down |
| DDIT4 | -1.34743 | 0.00195 | down | -1.84712 | <0.0001 | down |
| DENND1A | -1.21812 | 0.0177 | down | -7.54346 | <0.0001 | down |
| DGKI | 1.2915 | 0.0311 | up | -4.2421 | <0.0001 | down |
| DHRS3 | 1.72313 | 0.0044 | up | -1.67561 | <0.0001 | down |
| DISP1 | 1.41277 | 0.0043 | up | -5.40133 | <0.0001 | down |
| DNAJB14 | -1.13099 | 0.0032 | down | 2.299994 | <0.0001 | up |
| DSP | 1.39726 | <0.0001 | up | -2.51135 | <0.0001 | down |
| DUSP16 | -1.36995 | 0.01195 | down | -3.09115 | <0.0001 | down |
| ELK4 | -1.23976 | 0.00135 | down | 3.352835 | <0.0001 | up |
| ENPP1 | -1.05261 | 0.0026 | down | 6.425524 | <0.0001 | up |
| EPG5 | 1.04072 | <0.0001 | up | 2.731498 | <0.0001 | up |
| F11R | 3.55486 | 0.00635 | up | 2.197205 | <0.0001 | up |
| FADS2 | -1.52108 | 0.00035 | down | 2.489629 | <0.0001 | up |
| FAM129A | -1.90211 | <0.0001 | down | -2.01914 | <0.0001 | down |
| FAM156B | 3.6221 | 0.0052 | up | -1.94206 | <0.0001 | down |
| FAM83D | -2.2765 | 0.0391 | down | -2.06679 | <0.0001 | down |
| FBLN2 | -2.17884 | 0.0372 | down | -2.53148 | <0.0001 | down |
| FGD4 | 1.20475 | 0.01625 | up | 8.107286 | <0.0001 | up |
| FIGN | -1.17276 | 0.01145 | down | 9.709945 | <0.0001 | up |
| FNDC3A | -2.10452 | <0.0001 | down | 9.983421 | <0.0001 | up |
| GAB1 | -1.42174 | 0.0437 | down | 2.679617 | <0.0001 | up |
| GALNT4 | -1.41944 | 0.0066 | down | 3.452616 | <0.0001 | up |
| GEN1 | -2.23292 | 0.00025 | down | -8.60622 | <0.0001 | down |
| GPR85 | -3.34449 | <0.0001 | down | 4.118675 | <0.0001 | up |
| HDAC4 | -1.77314 | 0.029 | down | 2.913321 | <0.0001 | up |
| HERPUD1 | -1.85613 | <0.0001 | down | -1.32539 | <0.0001 | down |
| HIST1H2AE | Inf | <0.0001 | up | -1.8262 | <0.0001 | down |
| IBTK | -1.21508 | 0.0039 | down | 4.55111 | <0.0001 | up |
| IFNAR1 | -1.15402 | <0.0001 | down | 2.513844 | <0.0001 | up |
| IRS1 | -1.78678 | <0.0001 | down | -4.96935 | <0.0001 | down |
| ITGAV | -1.07455 | <0.0001 | down | 1.809838 | <0.0001 | up |
| KAT6A | -1.39347 | <0.0001 | down | -7.56758 | <0.0001 | down |
| KCTD15 | -1.18132 | 0.0026 | down | 4.770242 | <0.0001 | up |
| KIAA1324L | 3.91892 | 0.0467 | up | 8.010668 | <0.0001 | up |
| LATS1 | -1.32221 | <0.0001 | down | -1.55603 | <0.0001 | down |
| LGALS3BP | 2.08446 | <0.0001 | up | -1.59255 | <0.0001 | down |
| LONRF1 | -2.23969 | 0.0012 | down | 6.540279 | <0.0001 | up |
| LRCH3 | -2.08706 | 0.00075 | down | 4.501316 | <0.0001 | up |
| LRFN4 | 1.44555 | 0.03595 | up | 4.384856 | <0.0001 | up |
| MANEA | -1.66657 | <0.0001 | down | 3.226544 | <0.0001 | up |
| MAP1A | 1.26372 | <0.0001 | up | -2.31338 | <0.0001 | down |
| MAPK6 | -1.31604 | 0.00195 | down | -1.18992 | <0.0001 | down |
| MBD5 | -1.75944 | 0.03195 | down | -1.11512 | <0.0001 | down |
| MBNL2 | -1.2916 | <0.0001 | down | -1.46165 | <0.0001 | down |
| MCOLN2 | Inf | <0.0001 | up | 3.376184 | <0.0001 | up |
| MDC1 | -2.21301 | <0.0001 | down | -2.27368 | <0.0001 | down |
| MDFIC | -2.82706 | <0.0001 | down | 6.194242 | <0.0001 | up |
| MECOM | -1.32249 | 0.001 | down | 5.280834 | <0.0001 | up |
| MFHAS1 | -1.36732 | 0.01655 | down | 3.943073 | <0.0001 | up |
| MGA | -1.2449 | 0.001 | down | 5.947763 | <0.0001 | up |
| MIER2 | 1.12671 | 0.01125 | up | 3.032388 | <0.0001 | up |
| MIS18BP1 | -4.01406 | <0.0001 | down | 9.104337 | <0.0001 | up |
| MKI67 | -3.55421 | <0.0001 | down | -1.66764 | <0.0001 | down |
| MSL2 | -2.19404 | <0.0001 | down | -2.35406 | <0.0001 | down |
| MTDH | -1.25301 | 0.0211 | down | -6.12118 | <0.0001 | down |
| NAV1 | 1.01978 | 0.00015 | up | 4.23349 | <0.0001 | up |
| NBPF1 | -1.05081 | <0.0001 | down | -1.57527 | <0.0001 | down |
| NCEH1 | -1.22698 | 0.00075 | down | -2.02712 | <0.0001 | down |
| NCKAP5 | 1.22528 | 0.0162 | up | -3.23624 | <0.0001 | down |
| NFATC3 | -1.20137 | 0.00095 | down | -2.97347 | <0.0001 | down |
| NHSL1 | -2.07683 | 0.0239 | down | 3.666975 | <0.0001 | up |
| NPTXR | 1.54062 | 0.02965 | up | 5.597006 | <0.0001 | up |
| NRIP1 | -1.27934 | <0.0001 | down | -1.34181 | <0.0001 | down |
| NRP2 | -1.33269 | 0.0026 | down | -1.55247 | <0.0001 | down |
| NSL1 | -1.23755 | 0.0067 | down | 3.233768 | <0.0001 | up |
| PCDH18 | -2.18413 | <0.0001 | down | 5.032908 | <0.0001 | up |
| PCDH7 | -1.05627 | <0.0001 | down | 2.936646 | <0.0001 | up |
| PCNXL3 | 1.46776 | 0.00025 | up | 2.143537 | <0.0001 | up |
| PDE4D | -2.33151 | <0.0001 | down | -3.85783 | <0.0001 | down |
| PDGFRA | -1.08218 | <0.0001 | down | 1.54969 | <0.0001 | up |
| PDZRN3 | -1.03006 | 0.03315 | down | -1.98042 | <0.0001 | down |
| PGAP1 | -1.51002 | 0.00065 | down | 3.266103 | <0.0001 | up |
| PHF2 | -1.08973 | 0.00885 | down | -1.7768 | <0.0001 | down |
| PHF3 | -1.06196 | 0.04515 | down | 2.834778 | <0.0001 | up |
| PHLDA3 | 1.26046 | 0.002 | up | 2.143744 | <0.0001 | up |
| PIAS4 | 1.68189 | 0.0378 | up | 4.353743 | <0.0001 | up |
| PIK3R1 | -2.57005 | <0.0001 | down | 4.505379 | <0.0001 | up |
| PLAG1 | -1.15706 | 0.0059 | down | -4.90434 | <0.0001 | down |
| PLEC | 1.1758 | <0.0001 | up | 1.930044 | <0.0001 | up |
| PM20D2 | -1.5324 | <0.0001 | down | 8.761219 | <0.0001 | up |
| POLQ | -3.53631 | 0.03135 | down | 3.472006 | <0.0001 | up |
| POLR3B | -1.50056 | 0.02185 | down | -2.63079 | <0.0001 | down |
| PPP2R3A | -1.32966 | 0.00585 | down | -4.82941 | <0.0001 | down |
| PRDM1 | -1.84669 | 0.004 | down | -7.99227 | <0.0001 | down |
| PRKG1 | -1.43778 | 0.00055 | down | -7.01011 | <0.0001 | down |
| PTX3 | -2.22312 | <0.0001 | down | -2.9719 | <0.0001 | down |
| PUS7L | -1.14939 | 0.00395 | down | 6.459207 | <0.0001 | up |
| PVR | 1.00568 | <0.0001 | up | -1.21963 | <0.0001 | down |
| RABEP1 | -1.21279 | 0.0102 | down | -1.91798 | <0.0001 | down |
| RALGPS2 | -1.79515 | <0.0001 | down | 8.145677 | <0.0001 | up |
| RASGRF2 | -1.4843 | <0.0001 | down | 3.323143 | <0.0001 | up |
| RBM41 | -1.62564 | 0.00125 | down | -2.22647 | <0.0001 | down |
| RICTOR | -1.10356 | 0.02605 | down | -5.03073 | <0.0001 | down |
| RNASEH1 | -1.45089 | 0.0033 | down | 4.041719 | <0.0001 | up |
| RNF169 | -1.21228 | 0.0045 | down | 3.020214 | <0.0001 | up |
| RPP14 | -1.16773 | 0.0278 | down | 2.874664 | <0.0001 | up |
| SACS | -1.27197 | <0.0001 | down | 2.586994 | <0.0001 | up |
| SAMD9 | -2.21525 | <0.0001 | down | 2.367634 | <0.0001 | up |
| SERPINB9 | -1.40805 | 0.02425 | down | 7.070956 | <0.0001 | up |
| SETD1A | -1.07047 | 0.00965 | down | 2.110483 | <0.0001 | up |
| SLC25A24 | -1.13916 | <0.0001 | down | -2.93973 | <0.0001 | down |
| SLC7A11 | -1.77129 | <0.0001 | down | -7.4694 | <0.0001 | down |
| SLC7A5 | -2.10799 | <0.0001 | down | -4.94804 | <0.0001 | down |
| SLFN5 | -1.33297 | <0.0001 | down | -1.89443 | <0.0001 | down |
| SMCR8 | -1.80592 | 0.01815 | down | 3.235865 | <0.0001 | up |
| SOX4 | -1.80564 | <0.0001 | down | -1.27528 | <0.0001 | down |
| SPATA5 | -1.98505 | 0.001 | down | -5.46384 | <0.0001 | down |
| SPECC1 | -1.00234 | 0.03385 | down | 1.127506 | <0.0001 | up |
| SSX2IP | -2.08859 | <0.0001 | down | -2.68636 | <0.0001 | down |
| STAG1 | -1.18215 | 0.00015 | down | -6.42122 | <0.0001 | down |
| STK10 | 1.27619 | <0.0001 | up | 6.017398 | <0.0001 | up |
| SVEP1 | 1.54217 | <0.0001 | up | -1.38726 | <0.0001 | down |
| SYNCRIP | -1.57112 | 0.00665 | down | 2.291407 | <0.0001 | up |
| SYNE2 | -2.83455 | 0.00015 | down | 2.316317 | <0.0001 | up |
| SYNPO2 | 1.24052 | 0.01985 | up | 7.164572 | <0.0001 | up |
| TANC2 | 1.09595 | 0.0024 | up | 6.519035 | <0.0001 | up |
| TCERG1 | -1.29917 | 0.0011 | down | -5.33614 | <0.0001 | down |
| TEF | -1.08214 | 0.0427 | down | 3.192311 | <0.0001 | up |
| TENM3 | -1.31491 | 0.0061 | down | -4.22707 | <0.0001 | down |
| TET2 | -1.21147 | 0.002 | down | 7.079064 | <0.0001 | up |
| TFDP2 | -1.52834 | 0.01255 | down | 7.651769 | <0.0001 | up |
| TGFBR1 | -1.01885 | 0.00015 | down | -1.62363 | <0.0001 | down |
| THAP5 | -1.51954 | 0.0166 | down | 4.844283 | <0.0001 | up |
| THEMIS2 | -4.36911 | 0.04335 | down | 2.945147 | <0.0001 | up |
| TMTC2 | -2.02601 | 0.0217 | down | 8.497453 | <0.0001 | up |
| TNFAIP2 | -2.09103 | 0.00535 | down | 2.756898 | <0.0001 | up |
| TNFAIP3 | -1.28002 | 0.0297 | down | 4.076156 | <0.0001 | up |
| TNFAIP8 | -2.43917 | <0.0001 | down | -1.48179 | <0.0001 | down |
| TOR1AIP1 | -1.51803 | 0.00125 | down | 4.811136 | <0.0001 | up |
| UBTD2 | -1.20832 | 0.00015 | down | 3.90996 | <0.0001 | up |
| UNC5B | -2.9096 | 0.00065 | down | -1.02556 | <0.0001 | down |
| USP6NL | -1.04965 | 0.033 | down | -1.84588 | <0.0001 | down |
| VLDLR | -1.74178 | <0.0001 | down | -6.08026 | <0.0001 | down |
| VPS18 | 1.00609 | 0.00175 | up | -3.26596 | <0.0001 | down |
| WDR43 | -1.79289 | 0.00015 | down | -1.5233 | <0.0001 | down |
| WDR81 | 2.78266 | 0.01045 | up | 1.080333 | <0.0001 | up |
| WHSC1L1 | -1.67115 | <0.0001 | down | -1.54658 | <0.0001 | down |
| ZBTB10 | -5.06908 | 0.04865 | down | -6.90448 | <0.0001 | down |
| ZBTB38 | -1.35567 | <0.0001 | down | -1.84027 | <0.0001 | down |
| ZBTB41 | -1.00375 | 0.0148 | down | 4.492681 | <0.0001 | up |
| ZBTB6 | -2.0751 | 0.00025 | down | 2.636188 | <0.0001 | up |
| ZC3H12C | -1.04066 | 0.0089 | down | 2.770612 | <0.0001 | up |
| ZFHX4 | -1.20165 | <0.0001 | down | -3.15318 | <0.0001 | down |
| ZFP14 | -1.35652 | 0.01425 | down | 2.486126 | <0.0001 | up |
| ZFP62 | -1.95666 | <0.0001 | down | 5.253377 | <0.0001 | up |
| ZFP69B | -2.1462 | 0.02155 | down | -1.63622 | <0.0001 | down |
| ZMYM1 | -1.38356 | 0.00135 | down | -5.73306 | <0.0001 | down |
| ZNF621 | -1.89797 | <0.0001 | down | -1.24645 | <0.0001 | down |
| ZNF675 | -1.55281 | 0.00195 | down | 3.548588 | <0.0001 | up |
| ZSCAN12 | -1.75983 | 0.00205 | down | -2.20313 | <0.0001 | down |
| ZSCAN29 | -1.02966 | 0.01275 | down | -3.60853 | <0.0001 | down |

Note: up: upregulated genes (Log_2_^FC^ ≥ 1, *P* < 0.0001); down: downregulated genes (Log_2_^FC^ ≤ -1, *P* < 0.001).

Supplemental Table 2. The 198 differentially expressed genes with different m6A peaks in the PSp group.

| Gene | Gene expression | | | m6A | | |
| --- | --- | --- | --- | --- | --- | --- |
|  | Log_2_^FC^ | Regulation | *P* | Log_2_^FC^ | *P* | Regulation |
| ASPM | -3.93102 | down | <0.0001 | -3.58803 | <0.0001 | down |
| HJURP | -3.89466 | down | <0.0001 | 6.373223 | <0.0001 | up |
| SHCBP1 | -3.79383 | down | <0.0001 | 5.823273 | <0.0001 | up |
| MYBL2 | -3.68576 | down | 0.0021 | 8.157487 | <0.0001 | up |
| BIRC5 | -3.64644 | down | <0.0001 | 8.260397 | <0.0001 | up |
| CDCA5 | -3.55158 | down | <0.0001 | 8.484219 | <0.0001 | up |
| BLM | -3.33685 | down | 0.0041 | -8.20457 | <0.0001 | down |
| FOXM1 | -3.10803 | down | 0.0097 | 6.377154 | <0.0001 | up |
| SPAG5 | -2.95754 | down | 0.01685 | 6.246192 | <0.0001 | up |
| MKI67 | -2.94977 | down | 0.00115 | 6.086693 | <0.0001 | up |
| RAD54B | -2.83747 | down | 0.0066 | -9.07708 | <0.0001 | down |
| TPX2 | -2.80357 | down | <0.0001 | 6.786431 | <0.0001 | up |
| ZNF70 | -2.7707 | down | 0.00065 | 11.34291 | <0.0001 | up |
| ZNF713 | -2.72273 | down | 0.02015 | -11.9465 | <0.0001 | down |
| NRXN3 | -2.53664 | down | <0.0001 | -6.15831 | <0.0001 | down |
| TK1 | -2.5321 | down | <0.0001 | 8.559271 | <0.0001 | up |
| HIST1H2AG | -2.48487 | down | 0.0203 | 11.32705 | <0.0001 | up |
| CCNF | -2.47682 | down | <0.0001 | 8.531254 | <0.0001 | up |
| CENPF | -2.37006 | down | <0.0001 | 2.914599 | <0.0001 | up |
| ZFP69B | -2.30243 | down | 0.0154 | -9.97126 | <0.0001 | down |
| TMCC3 | -2.24313 | down | 0.0377 | -12.2545 | <0.0001 | down |
| SYNE2 | -2.14826 | down | 0.00785 | -8.48423 | <0.0001 | down |
| ATF7IP | -2.12505 | down | 0.02805 | -11.7583 | <0.0001 | down |
| ZWINT | -2.07402 | down | 0.00315 | 7.350939 | <0.0001 | up |
| PDSS2 | -2.03639 | down | 0.0412 | -3.71279 | <0.0001 | down |
| FUT10 | -2.03012 | down | 0.02835 | -3.39121 | <0.0001 | down |
| SGOL2 | -2.01358 | down | <0.0001 | -4.74444 | <0.0001 | down |
| INHBE | -2.00849 | down | 0.04365 | -10.8412 | <0.0001 | down |
| PODXL | -2.00433 | down | 0.00045 | 6.150824 | <0.0001 | up |
| KCNK2 | -1.98615 | down | <0.0001 | -11.4743 | <0.0001 | down |
| SOX11 | -1.91585 | down | <0.0001 | -6.90009 | <0.0001 | down |
| LCORL | -1.85523 | down | 0.00245 | -3.39057 | <0.0001 | down |
| CHAF1A | -1.82985 | down | 0.0192 | 4.605293 | <0.0001 | up |
| BTBD7 | -1.79684 | down | 0.0245 | 2.233904 | <0.0001 | up |
| PDCL | -1.77665 | down | 0.0453 | -2.70511 | <0.0001 | down |
| HERPUD1 | -1.7724 | down | <0.0001 | -5.42969 | <0.0001 | down |
| USP1 | -1.75527 | down | <0.0001 | -2.32898 | <0.0001 | down |
| GEN1 | -1.73767 | down | 0.0017 | -8.27612 | <0.0001 | down |
| APLF | -1.71656 | down | 0.00385 | -10.4447 | <0.0001 | down |
| GALNT4 | -1.68873 | down | 0.00455 | 7.411935 | <0.0001 | up |
| CDH2 | -1.68172 | down | <0.0001 | -5.07515 | <0.0001 | down |
| HS3ST3A1 | -1.66899 | down | 0.04825 | -8.67522 | <0.0001 | down |
| DBF4 | -1.65855 | down | 0.0052 | -2.64698 | <0.0001 | down |
| B3GNT5 | -1.58272 | down | 0.0011 | -2.84448 | <0.0001 | down |
| GPR85 | -1.58162 | down | 0.0389 | 6.797013 | <0.0001 | up |
| TWIST1 | -1.56947 | down | <0.0001 | -3.58912 | <0.0001 | down |
| PLA2G12A | -1.5664 | down | 0.0187 | -11.4406 | <0.0001 | down |
| POLR3B | -1.56283 | down | 0.018 | -6.20618 | <0.0001 | down |
| NET1 | -1.54717 | down | <0.0001 | 3.4327 | <0.0001 | up |
| SYT1 | -1.54369 | down | <0.0001 | -10.7688 | <0.0001 | down |
| LCOR | -1.51204 | down | 0.00075 | -6.54562 | <0.0001 | down |
| CCDC34 | -1.48669 | down | 0.01025 | 3.160804 | <0.0001 | up |
| ZFP62 | -1.46563 | down | 0.00595 | -4.14262 | <0.0001 | down |
| RTKN2 | -1.45253 | down | 0.0059 | -5.95571 | <0.0001 | down |
| WHSC1 | -1.41997 | down | 0.0071 | 4.845048 | <0.0001 | up |
| PTX3 | -1.34618 | down | <0.0001 | -4.45764 | <0.0001 | down |
| C5orf24 | -1.34243 | down | 0.01325 | -8.09752 | <0.0001 | down |
| C10orf12 | -1.32941 | down | 0.0065 | -4.39654 | <0.0001 | down |
| PPP2R3A | -1.32703 | down | 0.00635 | -7.79181 | <0.0001 | down |
| CEP78 | -1.32467 | down | <0.0001 | -7.38501 | <0.0001 | down |
| HN1L | -1.32418 | down | 0.00065 | 2.815728 | <0.0001 | up |
| CDC27 | -1.31486 | down | 0.00465 | -1.04481 | <0.0001 | down |
| ARRB2 | -1.30978 | down | 0.004 | 9.189275 | <0.0001 | up |
| RNF168 | -1.3087 | down | 0.0071 | -3.08855 | <0.0001 | down |
| GOLGA4 | -1.28184 | down | 0.0067 | -12.9652 | <0.0001 | down |
| SSR1 | -1.2673 | down | 0.0011 | 5.641527 | <0.0001 | up |
| TRMT13 | -1.25613 | down | 0.0112 | -6.08524 | <0.0001 | down |
| RFC2 | -1.24724 | down | 0.0156 | 11.15634 | <0.0001 | up |
| SIPA1L3 | -1.23868 | down | 0.0134 | 9.761136 | <0.0001 | up |
| NRP1 | -1.23745 | down | <0.0001 | -1.79965 | <0.0001 | down |
| MCM2 | -1.23313 | down | 0.0235 | 4.973251 | <0.0001 | up |
| GADD45B | -1.23118 | down | 0.04115 | -2.02762 | <0.0001 | down |
| RBMS3 | -1.23027 | down | 0.01485 | 6.710314 | <0.0001 | up |
| DOPEY2 | -1.21871 | down | 0.00075 | -5.81488 | <0.0001 | down |
| ASNSD1 | -1.21847 | down | 0.00115 | -9.7146 | <0.0001 | down |
| E2F1 | -1.20815 | down | 0.0224 | 7.538423 | <0.0001 | up |
| POP1 | -1.18436 | down | 0.0061 | 9.693016 | <0.0001 | up |
| SACS | -1.17878 | down | <0.0001 | -5.02621 | <0.0001 | down |
| MEIS2 | -1.14519 | down | 0.0107 | -3.8648 | <0.0001 | down |
| NFIC | -1.14041 | down | 0.00645 | 4.10534 | <0.0001 | up |
| NRIP1 | -1.13356 | down | <0.0001 | -12.2989 | <0.0001 | down |
| LMNB2 | -1.13272 | down | 0.03375 | 5.752489 | <0.0001 | up |
| NAA50 | -1.12516 | down | 0.0011 | 6.74685 | <0.0001 | up |
| TNC | -1.12508 | down | <0.0001 | -10.8931 | <0.0001 | down |
| CEP70 | -1.12203 | down | 0.0454 | -8.14643 | <0.0001 | down |
| SCN9A | -1.1211 | down | <0.0001 | -4.66421 | <0.0001 | down |
| OLFML3 | -1.10327 | down | <0.0001 | -8.45113 | <0.0001 | down |
| NCEH1 | -1.10195 | down | 0.0017 | 2.104432 | <0.0001 | up |
| BTBD11 | -1.08895 | down | 0.04825 | 10.44607 | <0.0001 | up |
| BRI3BP | -1.08226 | down | 0.03145 | 3.720784 | <0.0001 | up |
| RIF1 | -1.07656 | down | 0.0019 | -6.30695 | <0.0001 | down |
| SPTY2D1 | -1.06856 | down | 0.0454 | -4.80277 | <0.0001 | down |
| SLC7A5 | -1.04379 | down | 0.00015 | 2.98827 | <0.0001 | up |
| H2AFX | -1.04373 | down | 0.0239 | 4.268295 | <0.0001 | up |
| MEX3D | -1.03834 | down | 0.02115 | 2.112125 | <0.0001 | up |
| TCERG1 | -1.03315 | down | 0.0072 | -3.79437 | <0.0001 | down |
| EXOSC2 | -1.0278 | down | 0.0312 | 4.284312 | <0.0001 | up |
| FOXE1 | -1.02568 | down | 0.0205 | 7.303781 | <0.0001 | up |
| NR2F2 | -1.02004 | down | <0.0001 | -2.00992 | <0.0001 | down |
| SYNPO | 1.00813 | up | 0.00245 | -11.0409 | <0.0001 | down |
| PAK4 | 1.01475 | up | 0.03065 | 7.231412 | <0.0001 | up |
| IRS2 | 1.015 | up | <0.0001 | -2.77229 | <0.0001 | down |
| HEG1 | 1.02177 | up | <0.0001 | -11.9598 | <0.0001 | down |
| GALNT5 | 1.03281 | up | <0.0001 | -3.62068 | <0.0001 | down |
| ITGB8 | 1.03554 | up | 0.00655 | -4.25813 | <0.0001 | down |
| STARD3 | 1.04074 | up | 0.03305 | 2.918968 | <0.0001 | up |
| CEBPB | 1.0444 | up | 0.00025 | 4.258897 | <0.0001 | up |
| PLEKHO2 | 1.04496 | up | 0.0038 | -2.40288 | <0.0001 | down |
| C8orf4 | 1.04652 | up | 0.0252 | 10.7533 | <0.0001 | up |
| FAM198B | 1.05111 | up | 0.00285 | -9.27126 | <0.0001 | down |
| LUM | 1.06942 | up | 0.00015 | -14.2825 | <0.0001 | down |
| KLF9 | 1.07135 | up | 0.00825 | -6.77861 | <0.0001 | down |
| NID2 | 1.07221 | up | 0.00585 | -9.12386 | <0.0001 | down |
| WDR6 | 1.07543 | up | 0.04745 | 1.975869 | <0.0001 | up |
| CCNL2 | 1.07657 | up | <0.0001 | -1.72854 | <0.0001 | down |
| MEGF9 | 1.07854 | up | <0.0001 | 5.948367 | <0.0001 | up |
| ARHGAP24 | 1.09176 | up | 0.0061 | -9.98085 | <0.0001 | down |
| ZCCHC14 | 1.09604 | up | 0.00035 | -9.63408 | <0.0001 | down |
| TSKU | 1.09668 | up | 0.02075 | 2.876755 | <0.0001 | up |
| CCDC127 | 1.10667 | up | 0.0263 | 3.051933 | <0.0001 | up |
| SEMA6D | 1.112 | up | 0.0025 | -10.8389 | <0.0001 | down |
| ALDH3A1 | 1.11297 | up | 0.0217 | 11.34369 | <0.0001 | up |
| CYP1B1 | 1.12661 | up | 0.0033 | -3.5428 | <0.0001 | down |
| SLITRK5 | 1.12858 | up | 0.00165 | -3.72441 | <0.0001 | down |
| ABCC1 | 1.12936 | up | <0.0001 | 4.498251 | <0.0001 | up |
| SLC16A3 | 1.13028 | up | 0.00365 | -3.57579 | <0.0001 | down |
| SYNGR2 | 1.13653 | up | 0.0113 | 4.519578 | <0.0001 | up |
| PHLDA3 | 1.16272 | up | 0.00425 | 4.107403 | <0.0001 | up |
| SCAMP4 | 1.16272 | up | 0.0414 | 10.59283 | <0.0001 | up |
| SLC25A29 | 1.16283 | up | 0.0332 | 2.936022 | <0.0001 | up |
| MAFG | 1.18156 | up | 0.00565 | 4.409324 | <0.0001 | up |
| PLEKHA6 | 1.1836 | up | 0.0408 | 12.07267 | <0.0001 | up |
| MAFK | 1.19175 | up | <0.0001 | 3.838182 | <0.0001 | up |
| ASAP3 | 1.19697 | up | 0.0051 | -10.0108 | <0.0001 | down |
| C1R | 1.20617 | up | <0.0001 | -2.76871 | <0.0001 | down |
| NDN | 1.21002 | up | 0.01615 | -9.59615 | <0.0001 | down |
| TSPO | 1.22107 | up | 0.0308 | -8.67842 | <0.0001 | down |
| IGFBP7 | 1.26092 | up | 0.01315 | -3.6365 | <0.0001 | down |
| PCNXL3 | 1.29203 | up | 0.0015 | 2.934847 | <0.0001 | up |
| RERE | 1.34371 | up | 0.0088 | -7.86476 | <0.0001 | down |
| LRP1 | 1.36865 | up | <0.0001 | -5.12833 | <0.0001 | down |
| LGALS3BP | 1.38738 | up | <0.0001 | -2.5454 | <0.0001 | down |
| SMOX | 1.39138 | up | 0.0285 | 10.49738 | <0.0001 | up |
| TRANK1 | 1.40744 | up | 0.0061 | -7.92184 | <0.0001 | down |
| ASAH1 | 1.42251 | up | <0.0001 | -1.70945 | <0.0001 | down |
| DISP1 | 1.42604 | up | 0.00115 | -6.98185 | <0.0001 | down |
| ULK1 | 1.43595 | up | 0.0016 | 3.222707 | <0.0001 | up |
| MUC5B | 1.46567 | up | 0.04125 | 12.83342 | <0.0001 | up |
| GAS2L1 | 1.46802 | up | 0.0144 | 4.080722 | <0.0001 | up |
| MANBA | 1.50656 | up | <0.0001 | -3.11506 | <0.0001 | down |
| CLCF1 | 1.50953 | up | 0.01875 | 9.483872 | <0.0001 | up |
| LDB1 | 1.52589 | up | 0.0041 | -2.71258 | <0.0001 | down |
| PPP1R3C | 1.54475 | up | <0.0001 | -3.59147 | <0.0001 | down |
| ASB13 | 1.59712 | up | 0.00895 | 2.771698 | <0.0001 | up |
| NCKAP5 | 1.59958 | up | 0.0176 | -4.88577 | <0.0001 | down |
| SNED1 | 1.62648 | up | 0.0317 | -1.55841 | <0.0001 | down |
| DGKI | 1.62998 | up | 0.00055 | -8.40131 | <0.0001 | down |
| TRIM16L | 1.64535 | up | 0.0083 | 6.956791 | <0.0001 | up |
| C1S | 1.72858 | up | <0.0001 | -3.74071 | <0.0001 | down |
| PLXDC2 | 1.82673 | up | <0.0001 | -9.02265 | <0.0001 | down |
| HERC1 | 1.86644 | up | 0.01515 | -7.11483 | <0.0001 | down |
| PKDCC | 1.91044 | up | <0.0001 | 3.015435 | <0.0001 | up |
| TFAP2A | 1.9329 | up | 0.021 | 5.393696 | <0.0001 | up |
| HIST1H1C | 1.94654 | up | 0.00085 | 3.529158 | <0.0001 | up |
| OSGIN1 | 1.95743 | up | 0.0125 | 9.555175 | <0.0001 | up |
| GAS1 | 1.96401 | up | 0.00745 | -8.17026 | <0.0001 | down |
| PTGS2 | 1.96822 | up | 0.0011 | -6.36888 | <0.0001 | down |
| RNPEPL1 | 1.9726 | up | 0.02015 | 7.206526 | <0.0001 | up |
| CHPF | 1.98103 | up | <0.0001 | -2.28604 | <0.0001 | down |
| SIK1 | 2.00499 | up | 0.00265 | 2.193555 | <0.0001 | up |
| PGPEP1 | 2.09787 | up | 0.0018 | -7.76044 | <0.0001 | down |
| ARHGAP26 | 2.15287 | up | 0.00015 | -8.13083 | <0.0001 | down |
| SLC40A1 | 2.18039 | up | <0.0001 | -6.82909 | <0.0001 | down |
| FIBIN | 2.20439 | up | 0.00035 | -12.5449 | <0.0001 | down |
| ANGPTL2 | 2.28737 | up | <0.0001 | -4.31265 | <0.0001 | down |
| ENDOV | 2.30374 | up | <0.0001 | 2.868847 | <0.0001 | up |
| HIST2H2BE | 2.40939 | up | <0.0001 | 4.854794 | <0.0001 | up |
| SLITRK6 | 2.41594 | up | <0.0001 | 10.22762 | <0.0001 | up |
| IGSF10 | 2.41961 | up | 0.0418 | -7.15583 | <0.0001 | down |
| UNC13D | 2.48385 | up | 0.00045 | 8.498074 | <0.0001 | up |
| RDH10 | 2.52905 | up | 0.0296 | -9.15127 | <0.0001 | down |
| TNFRSF21 | 2.56222 | up | <0.0001 | 10.07606 | <0.0001 | up |
| IL16 | 2.56235 | up | 0.0022 | -9.15051 | <0.0001 | down |
| WDR81 | 2.56482 | up | 0.0122 | 2.081838 | <0.0001 | up |
| PTGFRN | 2.61104 | up | 0.0025 | -2.65932 | <0.0001 | down |
| DAPK1 | 2.68192 | up | <0.0001 | 1.765273 | <0.0001 | up |
| MX1 | 2.85913 | up | 0.0467 | 2.875423 | <0.0001 | up |
| EVA1C | 2.94158 | up | <0.0001 | 9.223362 | <0.0001 | up |
| GGT1 | 2.95178 | up | 0.00015 | -4.2614 | <0.0001 | down |
| FRY | 2.99046 | up | <0.0001 | -3.18538 | <0.0001 | down |
| SVEP1 | 3.07447 | up | <0.0001 | -3.56107 | <0.0001 | down |
| ADAMTS19 | 3.21452 | up | 0.01175 | 9.488844 | <0.0001 | up |
| RGS2 | 3.21467 | up | <0.0001 | -9.78637 | <0.0001 | down |
| SLC12A8 | 3.54434 | up | <0.0001 | -9.31899 | <0.0001 | down |
| SCG2 | 3.66056 | up | 0.00175 | -12.0269 | <0.0001 | down |
| CCND2 | 3.72775 | up | <0.0001 | -9.64926 | <0.0001 | down |
| GAL3ST1 | 3.76319 | up | <0.0001 | 10.75664 | <0.0001 | up |
| PODNL1 | 3.82971 | up | <0.0001 | -9.90794 | <0.0001 | down |

Note: up: upregulated genes (Log_2_^FC^ ≥ 1, *P* < 0.0001); down: downregulated genes (Log_2_^FC^ ≤ -1, *P* < 0.001).

**
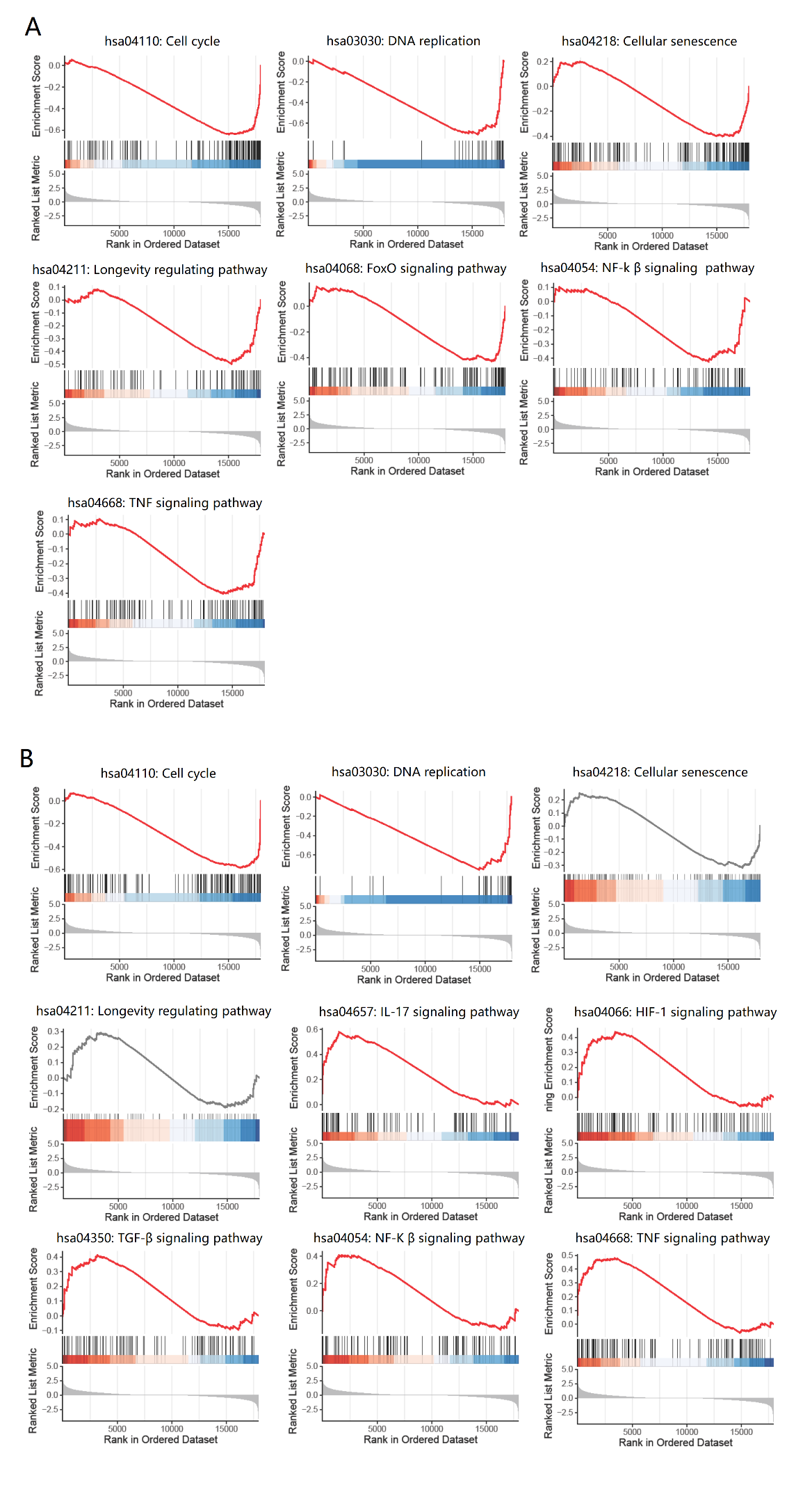
**

**Supplementary Figure 1.** **GSEA visualisation results**

(A) 49PDL vs. 22PDL; (B) PSp vs. 22PDL.


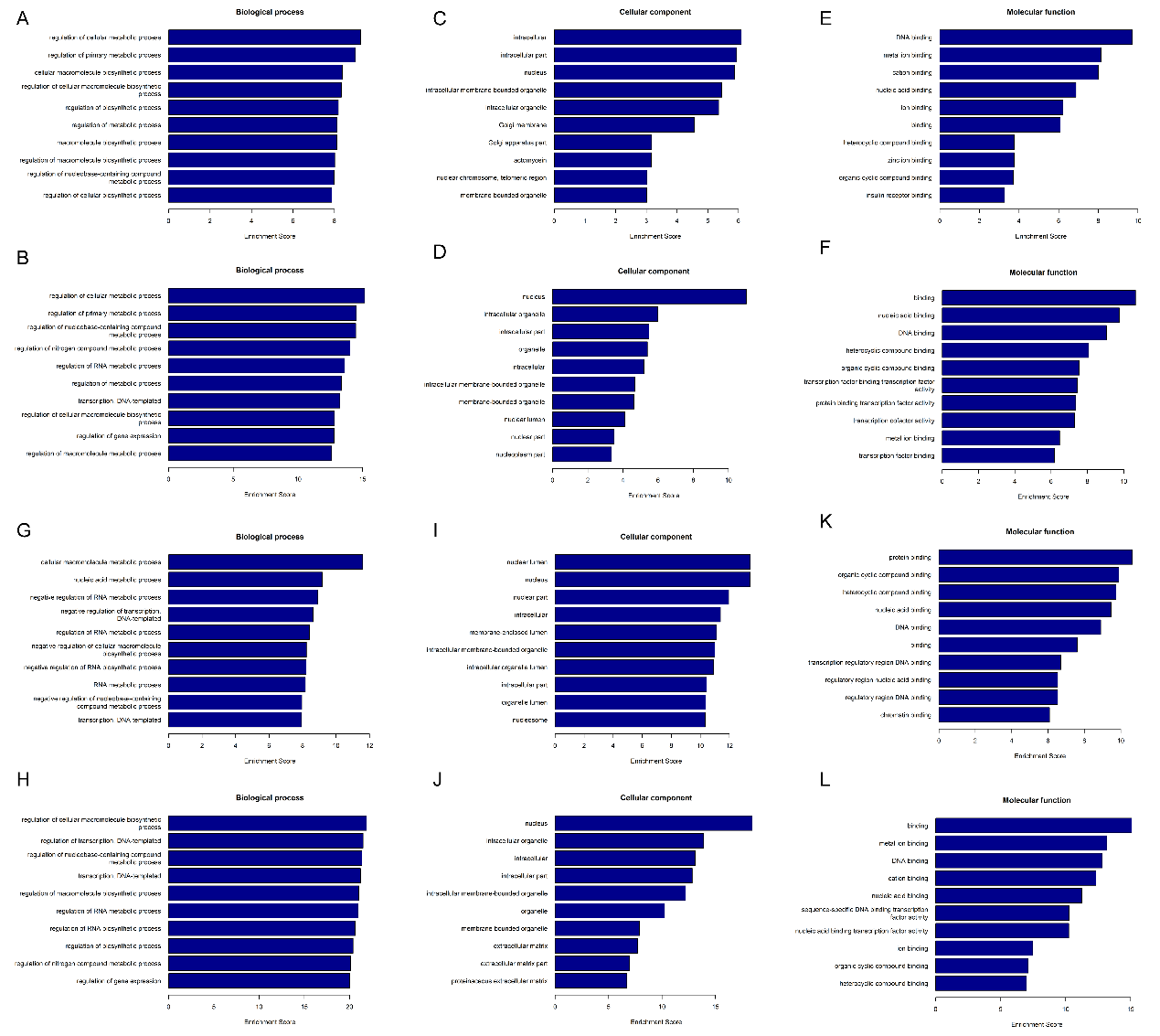


**Supplementary Figure 2.** **Replication of the top 10 most significantly enriched BP, CC, and MF in m6A-regulated genes in senescence.**

**(**A, C, E) 49PDL vs. 22PDL GO enrichment of m6A methylation up-regulated genes; (B, D, F) 49PDL vs. 22PDL GO enrichment of genes down-regulated by m6A methylation; (G, I, K) PSp vs. 22PDL GO enrichment of m6A methylation up-regulated genes; (H, J, L) PSp vs. 22PDL GO enrichment of genes down-regulated by m6A methylation.

**
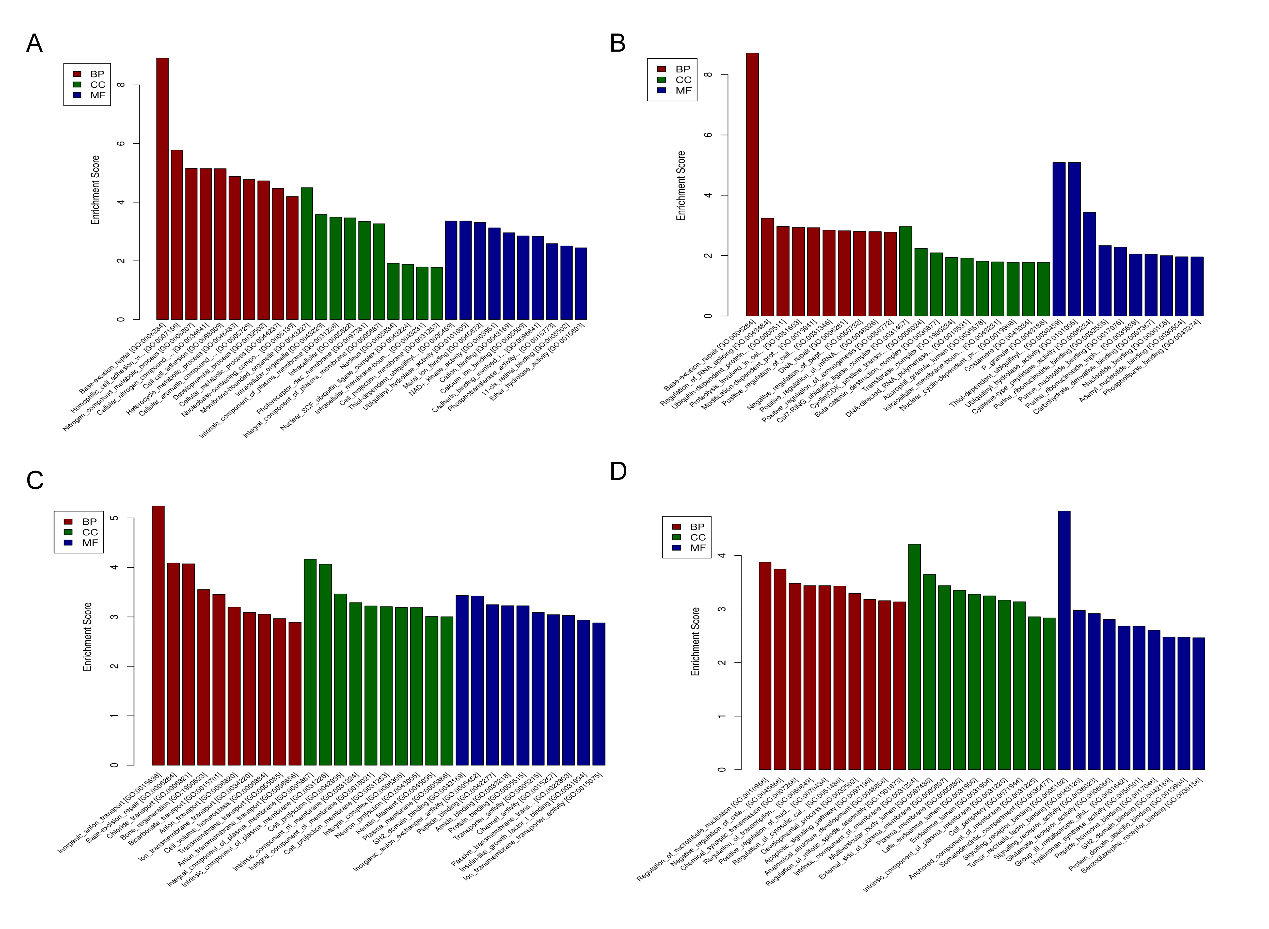
**

**Supplementary Figure 3. The top 10 most significantly enriched BPs, CCs and MFs among genes of 5mC peaks in senescence.**

(A-B) 49PDL vs. 22PDL; (C-D) PSp vs. 22PDL.
